# Genomic and Phenotypic Evaluation of the Gliadin-Degrading Probiotic *Bacillus amyloliquefaciens* EG025 from Cheonggukjang for Celiac Disease Treatment

**DOI:** 10.1007/s12602-025-10728-7

**Published:** 2025-09-02

**Authors:** Jinchul Jo, Seoae Cho, Heebal Kim

**Affiliations:** 1 Department of Agricultural Biotechnology and Research Institute of Agriculture and Life Sciences, Seoul National University, Room 5219, Building 200, Gwanak-Ro, Gwanak-Gu, Seoul, 08826 Republic of Korea; 2 eGnome Inc, Seoul, Republic of Korea

**Keywords:** *Bacillus amyloliquefaciens*, Celiac disease, Cheonggukjang, Gluten, Probiotics, Whole genome sequence

## Abstract

*Bacillus amyloliquefaciens* EG025 was isolated from cheonggukjang, a traditional Korean fermented soybean paste. Among multiple isolates from diverse fermented foods, *B.* *amyloliquefaciens* EG025 uniquely exhibited potent gliadin-degrading activity, a trait with the potential to attenuate the immunogenicity of gluten peptides implicated in celiac disease. *B.* *amyloliquefaciens* EG025 maintained robust enzymatic activity across a wide pH spectrum (neutral to alkaline) and, remarkably, retained over 90.4% of its maximal activity following a 2-h exposure to pH 2. In addition to its gliadin-degrading capability, *B.* *amyloliquefaciens* EG025 demonstrated exceptional acid and bile tolerance, indicating a strong capacity to survive and function under the harsh conditions of the human gastrointestinal tract. Whole-genome sequencing and comprehensive annotation revealed biosynthetic gene clusters for secondary metabolites known to confer host benefits, alongside key probiotic functional genes involved in gliadin degradation, acid and bile resistance, stress response, mucosal adhesion, and de novo vitamin biosynthesis. Importantly, safety assessment confirmed the absence of concerning antibiotic resistance determinants or virulence factors, underscoring the strain’s safety profile. Phylogenetic reconstruction further highlighted the genetic distinctiveness of EG025 relative to other *B. amyloliquefaciens* strains, emphasizing its novelty within the species. Collectively, these combined in vitro assay, genomic, and phylogenetic results underscore the significant probiotic potential of *B. amyloliquefaciens* EG025 and support its future development as a novel therapeutic candidate for improving gluten tolerance and managing celiac disease. Its safety and multifunctional attributes position it as a promising ingredient for functional foods targeted at gluten-sensitive populations.

**Supplementary Information:**

The online version contains supplementary material available at 10.1007/s12602-025-10728-7.

## Introduction

Celiac disease is a chronic autoimmune disorder triggered by the ingestion of gluten which is a complex of proteins predominantly found in wheat, barley, and rye. In genetically susceptible individuals, gluten-derived peptides, particularly those from gliadin, are allowed to cross the intestinal barrier, where an immune reaction is incited. As a result, inflammation, villous atrophy, and malabsorption are observed, leading to various gastrointestinal and systemic symptoms [1]. Although a strict gluten-free diet is regarded as the only effective treatment Although the gluten‐free diet remains the primary treatment for celiac disease, it imposes significant socio-economic burdens and often fails to meet patients’ nutritional needs, leading to deficiencies in fiber, vitamins, and minerals. Moreover, the high cost and variable quality of gluten‐free products leave them vulnerable to inadvertent contamination. The stringent restrictions of a gluten‐free diet can also diminish patients’ psychosocial well‐being by limiting food choices and complicating social activities. Collectively, these challenges highlight the necessity of complementary strategies to improve dietary safety, nutritional balance, and quality of life for individuals with celiac disease [2, 3].

One promising strategy is represented by the use of probiotics, which are believed to confer health benefits on the host when administered in adequate amounts. In the context of celiac disease, certain probiotic strains have been suggested to degrade gliadin, thereby diminishing the formation of harmful peptides [4]. For instance, a probiotic formulation consisting of *L. plantarum* DSM 15312 and *L. paracasei* DSM 13434 was administered to 40 children with celiac disease autoimmunity for six months, resulting in improvements in immune response markers (tTGA) even though they were instructed to continue a gluten-containing diet, suggesting a potential therapeutic effect in celiac disease [5]. Furthermore, it is thought that probiotics may modulate the gut microbiota, enhance the integrity of the intestinal barrier, and reduce inflammation, factors that are critical for effective disease management [6].

Since probiotics exert their effects by adhering to the host’s gastrointestinal tract, it is important that gliadin-degrading activity is well maintained even under extreme conditions such as those in the digestive system, and that the probiotic demonstrates sufficient survival during the digestive process [7]. Another critical aspect is evaluating whether the probiotic can safely colonize the host’s gut without causing any negative health effects. Recently, concerns such as the spread of antibiotic resistance by probiotics and documented cases of sepsis associated with their use have received significant attention, making it essential to thoroughly assess the safety of potential probiotic bacteria [8, 9]. Finally, because probiotics are inherently beneficial to host health, it is necessary to determine whether they can directly produce compounds that promote health or secrete antibacterial substances that form a protective barrier against pathogenic bacteria in the intestine, thereby contributing to immunomodulation [10].

Among the various probiotic candidates, *Bacillus spp.* have attracted considerable attention due to their unique physiological traits. These Gram-positive, spore-forming bacteria are renowned for their robustness, which enables them to withstand high temperatures, low pH, and bile salts, stresses commonly encountered during food processing and within the human gastrointestinal tract. Their ability to form endospores is considered to ensure survival under harsh conditions, rendering them well suited for incorporation into functional foods and therapeutic applications [11, 12]. Many *Bacillus* species, including *B. subtilis*, *B. coagulans*, and *B. amyloliquefaciens*, are recognized as GRAS (Generally Recognized as Safe), a designation by the U.S. Food and Drug Administration based on scientific evidence and a long history of safe consumption that indicates these organisms pose no significant risk under their intended conditions of use, thereby reinforcing their safety and suitability for use in food products and probiotic formulations [13–15]. In addition, a range of antimicrobial compounds such as bacteriocins, lipopeptides, and polyketides are produced by these bacteria, which help to suppress pathogenic microorganisms and promote a balanced intestinal microbiota [16].

While most previous studies have relied exclusively on in vitro assays, such as enzyme activity measurements, acid and bile tolerance tests, and antimicrobial screens, to evaluate probiotic candidates, these methods provide limited insight into the genetic basis of observed phenotypes and can be both labor-intensive and time-consuming [17]. By integrating whole-genome sequencing with classical laboratory assays, it becomes possible to rapidly annotate genes encoding proteolytic enzymes, bile salt hydrolases, stress-response proteins, and bacteriocin biosynthetic clusters. This combined approach not only accelerates the discovery process by prioritizing strains with defined mechanistic profiles but also enhances safety assessment through early detection of mobile genetic elements, such as antibiotic resistance genes and virulence factors [18, 19].

In this study, *Bacillus amyloliquefaciens* EG025 was isolated from cheonggukjang, a traditional Korean fermented soybean paste, and the strain was selected as a gliadin-degrading probiotic candidate. The effects of pH on its gliadin-degrading activity, as well as the residual activity after exposure to a range of pH conditions, were evaluated. Acid and bile tolerance were assessed to determine its viability as a probiotic, and its safety was further confirmed through both phenotypic and genotypic analyses. Whole-genome sequencing was also performed to characterize the structural features of the *B. amyloliquefaciens* EG025 genome and to identify probiotic-related functional genes and gene clusters involved in the biosynthesis of health-promoting bioactive compounds. In addition, a phylogenetic analysis based on whole-genome sequences was conducted to evaluate the genetic novelty of the strain. Collectively, this study integrates both experimental and genomic approaches to provide strong evidence for the probiotic potential of *B. amyloliquefaciens* EG025 and its possible application as a therapeutic agent for individuals with celiac disease.

## Materials and Methods

### Sample Collection and Probiotic Bacteria Isolation

Twenty-three Korean traditional fermented food samples, including kimchi, jeotgal, and cheonggukjang, were purchased from Bongcheon traditional market (Gwanak-gu, Seoul, South Korea) and Daerim central market (Yeongdeungpo-gu, Seoul, South Korea). To isolate a wide range of probiotic bacteria including lactic acid bacteria and *Bacillus* spp., each sample was serially diluted in 30 mL of PBS (pH 7.0) and plated onto deMan Rogosa Sharpe (MRS) agar or MRS-Cys agar media (BD Difco, USA), followed by incubation at 30 °C or 37 °C for three days under both aerobic and anaerobic conditions [20, 21]. Colonies exhibiting distinct morphological characteristics were selected and streaked onto fresh MRS agar plates for isolation. Single colonies were subsequently inoculated into 12 ml of MRS broth and cultured at 37 °C for two days. After incubation, each cultural broth was utilized for experimental purposes, and the remaining culture was centrifuged at 4,000 rpm for 10 min at 4 °C to harvest bacterial cells. Harvested cells were resuspended in 20% glycerol solution and stored at −80°C for preservation. Species identification of isolated bacterial strains was performed by sequencing the 16S rRNA gene (Cosmogenetech, Korea). To screen for isolates capable of degrading gliadin, each bacterial strain was streaked onto tryptic soy agar (TSA, BD Difco, USA) containing 20 g/L of gliadin. These plates were incubated at 37 °C for 2 days, and strains that produced a halo around the colonies were selected as possessing gliadin-degrading activity [22].

### Effect of pH on the Gliadin-Degrading Activity of *B. amyloliquefaciens* EG025

The gliadin-degrading activity of *B. amyloliquefaciens* EG025 and its changes according to pH were measured following the method described by Jaglan et al., with slight modifications [22]. First, TSA media were adjusted to pH values ranging from 3 to 10 using 1 M HCl or NaOH, and subsequently autoclaved. After autoclaving, the media were allowed to cool to room temperature. Once sufficiently cooled, 2 g of UV-treated gliadin was added to 100 mL of pH-adjusted TSA medium to prepare TSA-gliadin agar plates at different pH levels. Next, a single colony of *B. amyloliquefaciens* EG025 was inoculated into 20 mL of Luria–Bertani (LB, BD Difco, USA) broth and incubated at 37 °C with shaking at 180 rpm for 2 days. After incubation, the culture was harvested using 0.9% NaCl by centrifugation at 4,000 rpm for 10 min at 4 °C, and the cell suspension was adjusted to 6 × 10⁸ CFU/mL. Then, 2 μL of this cell suspension (approximately 1.2 × 10^6^ CFU) was spotted onto each of the TSA- gliadin plates and incubated at 37 °C for 48 h. The gliadin-degrading activity at different pH levels was assessed by measuring the diameter of the halo zones formed at 24 and 48 h. To evaluate the residual gliadin-degrading activity across the different pH conditions, 100 μL of the cell suspension (6 × 10⁸ CFU/mL) was mixed with 1.6 mL of buffers adjusted to the respective pH values (pH 2–5; 50 mM sodium acetate buffer, pH 6–8; 50 mM KH_2_PO_4_-K_2_HPO_4_ buffer, pH 9–10; 50 mM glycine-KCl-KOH buffer, pH 11–12; 50 mM K_2_HPO_4_-K_3_PO_4_ buffer) and incubated at room temperature with shaking at 180 rpm for 2 h. Following this incubation, each cell suspension was harvested by centrifugation at 6,000 rpm for 5 min at 4 °C, and the cells were washed twice and subsequently recovered in 100 μL of PBS pH 7.0. A 2 μL of recovered cell suspension was spotted onto TSA-gliadin plates (without pH adjustment) and incubated at 37 °C for 48 h. Finally, the residual gliadin-degrading activity was determined by measuring the diameters of the halo zones, as described above.

### Acid and Bile Tolerance Evaluation for *B. amyloliquefaciens* EG025

The acid and bile tolerance tests were performed with slight modifications to the method described by Dikbas et al. [23].To evaluate the acid resistance of *B. amyloliquefaciens* EG025, a simulated gastric solution was prepared. This solution was formulated by adding 3 mg/mL pepsin (Glentham, England, UK) to PBS adjusted to pH values (pH 2–4), and then filtered through a 0.45-μm syringe filter. Next, the cultural broth grown in LB media at 37 °C and 180 rpm for 2 days was inoculated to each 30 mL of the simulated gastric solution at a 3% (v/v), and the mixture was then incubated at 37 °C with shaking at 120 rpm for 6 h. At 2-h intervals, 200 μL samples were collected, serially diluted, and 100 μL of each dilution was spread onto LB agar plates. These plates were incubated at 37 °C for 2 days, and the colony-forming units (CFUs) were counted to calculate survival. The evaluation of bile resistance was conducted using a similar procedure, except that a bile solution was prepared by supplementing PBS (pH 7.0) with 0.3–1% oxgall (BD Difco, USA) instead of the simulated gastric solution. Under these conditions, the culture was incubated for 12 h. As before, 200 μL samples were collected every 2 h, serially diluted, and spread onto LB agar plates for CFU determination. Survival rate was calculated using the following formula based on the CFU/mL at the start of incubation (C_0_) and at each sampling time (C_t_).

$$Survival\;rate\left(\%\right)=\frac{CFU/mL\;at\;each\;time\;point\;(C_t)}{CFU/mL\;at\;start\;of\;incubation(C_0)}\times100$$

### Whole Genome Sequencing, Assembly and Annotation

For whole-genome sequencing, *B. amyloliquefaciens* EG025 was cultivated in LB broth at 37 °C and 180 rpm for 2 days. Genomic DNA extraction was performed using the PureHelix™ Genomic DNA Prep Kit (NanoHelix, Korea), and quality of the extracted DNA was confirmed via gel electrophoresis along with the 260/280 nm absorbance ratio measurement. The DNA was subsequently sheared to approximately 20 kb using a g-Tube (Covaris, USA), and fragments shorter than 10 kb were removed. Library preparation included steps for end-repair, nick repair, and Nanopore adapter ligation; purification was carried out with AMPure XP beads (Beckman Coulter, USA), and the library was quantified using the Quant-iT™ PicoGreen™ assay (Invitrogen, USA). Considering that bacterial genomes often contain repetitive elements and GC-rich regions that are challenging to resolve with short-read technologies, nanopore sequencing selected to achieve high-quality de novo assembly and complete genome closure [24]. Sequencing was conducted on a MinION flow cell (FLO-FLG001, R9.4.1) with MinION MIN-101B and MinKNOW software (version 19.12.5). Basecalling was performed using Guppy Basecaller v4.0.11, filtering out reads with an average Phred quality score below 7, and Porechop v0.2.4 (https://github.com/rrwick/Porechop) was employed to remove sequencing artifacts and chimeric reads. Nanofilt v2.7.1 was used to filter out reads with quality scores below 10 [25]. De novo genome assembly was conducted using Canu v2.1.0 with the parameter genomeSize = 3.9m, and the accuracy of the resulting contigs was improved by two rounds of Racon v1.4.3 followed by eight rounds of Medaka v1.0.3 (https://github.com/nanoporetech/medaka) under default conditions [26, 27]. Finally, the polished contigs were circularized using Circulator v1.5.5 with the parameters “–merge_min_id 85” and “–merge_breaklen 1000” [28]. Assembly quality was assessed using BUSCO v5.8.0 with the bacteria_odb10 dataset [29], and gene annotation was performed using Prokka v1.14.5 [30].

### Functional and Comparative Genomic Analysis

GenoVi v0.2.16 was employed to perform cluster of orthologous groups of proteins (COG) analysis and to generate a circular genome map, thereby elucidating gene functions and the organization of the genome [31]. Furthermore, antiSMASH v7.1.0 were used to detect gene clusters associated with the biosynthesis of valuable secondary metabolites [32]. To predict the presence of probiotic‐related genes within the genome of *B. amyloliquefaciens* EG025, gene sequences previously reported in scientific literature to be associated with probiotic traits in *Bacillus* spp. (including gliadin degradation, acid and bile tolerance, stress response, adhesion and aggregation, and vitamin biosynthesis) were collected. Based on this information, the corresponding CDS were retrieved from GenBank and UniProt and compiled into a single FASTA file. These sequences were then used to construct a custom BLAST database using the “makeblastdb -dbtype nucl” command provided by BLAST +. tBLASTn was then performed using this custom BLAST database and the peptide sequences generated from Prokka. Only hits with an e-value of ≤ 1e-5 and identity and coverage values of at least 75% were considered for functional annotation. For the comparative analysis of COG distribution and phylogenetic relationships, complete genome sequences of 102 *B. amyloliquefaciens* strains (assembled at the “chromosome” level or higher) were retrieved from the NCBI genome database. Together with the genome of EG025, a total of 103 strains were analyzed using Genovi with default parameters to calculate the percentage frequency of each COG category. The frequency data for each COG category were normalized across strains using z-score transformation, and a heatmap was generated using R software to visualize the resulting distribution patterns. For phylogenetic inference, a whole genome-based phylogenetic tree was constructed using CVTree v3.0.0 (InterList method, k = 7) in combination with MEGA-X, with *Priestia megaterium* ATCC 14581 designated as the outgroup [33]. In addition, average nucleotide identity (ANI) values among the strains were calculated using PyANI v0.3.0 with the ANIm method to further assess genomic relatedness [34].

### Safety Assessment for *B. amyloliquefaciens* EG025

The hemolytic activity of *Bacillus amyloliquefaciens* EG025 was assessed according to Buxton's protocol [35]. Initially, the strain was cultured in LB broth at 37 °C for 2 days. Following cultivation, the cultural broth was streaked onto sheep blood agar plates (MBcell, KisanBio, Korea) and incubated at 37 °C for 2 days. For comparative purposes, *Staphylococcus aureus* was grown in LB broth under the same conditions and then streaked onto sheep blood agar. Hemolytic activity was determined by observing the zones surrounding the colonies, where a green zone was indicative of α-hemolysis, a clear zone signified β-hemolysis, and the absence of any zone denoted γ-hemolysis.

For phenotypic antibiotic resistance testing, the antibiotics were selected with the highest defined daily dose per 1,000 inhabitants per day (DDD/1,000 person/days) in Korea, as reported in the 2016 National Health Insurance Service Research Report (Report number: 2016–20-001). These were grouped into nine categories: Penicillins (Amoxicillin–Clavulanate, Amoxicillin, Amoxicillin–Sulbactam), Cephems (Cefaclor, Cephradine, Cefpodoxime), Carbapenem (Meropenem), Monobactam (Aztreonam), Macrolides (Clarithromycin, Roxithromycin), Quinolones (Ciprofloxacin, Ofloxacin), Tetracyclines (Doxycycline, Minocycline), Aminoglycoside (Ribostamycin), and Rifamycin [36]. Phenotypic antibiotic resistance was evaluated using the Kirby-Bauer disk diffusion method. *B. amyloliquefaciens* EG025 was cultured in LB broth at 37 °C for 2 days. The cells were harvested by centrifugation, washed with saline, and standardized to a 0.5 McFarland turbidity (approximately 1.5 × 10^8^ CFU/mL). The resulting suspension was spread onto Iso-Sensitest agar plates (BD Difco, USA) using sterile cotton swabs, and antibiotic disks (Liofilchem, Italy) were applied. The plates were then incubated aerobically at 37 °C for 2 days. Zones of inhibition were measured and interpreted in accordance with Performance Standards for Antimicrobial Disk Susceptibility Tests [22].

In addition, to assess the genomic-level safety of strain EG025, a screening was conducted for antibiotic resistance genes and virulence factors using the Abricate software v1.0.1 (https://github.com/tseemann/ABRicate). Six antibiotic resistance gene databases (ARG-ANNOT, CARD, MEGARES, NCBI, PlasmidFinder, and ResFinder) and three virulence factor databases (EcOH, Ecoli_VF, and VFDB) were employed. The minimum identity and minimum coverage parameters were set at 80% and 70%, respectively, in accordance with EFSA guidelines [37]. To further validate the safety of EG025 as indicated by the Abricate results, comparative analyses were performed under identical conditions using the genomes of *B. amyloliquefaciens* LFB112 and TL106, probiotic strains previously reported to be safe in animal studies [38, 39].

### Statistical Analysis

For the experiments investigating changes in gliadin-degrading activity and the residual effects at each pH, one-way ANOVA followed by Dunnett’s post-hoc test was used to determine the significance of differences in activity at each pH compared to maximum activity. In the acid and bile tolerance evaluations, two-way ANOVA with Tukey-HSD post hoc analysis was performed to assess the significance of differences in survival across varying pH or bile concentrations at each time point.

### Genome Accession Number

The genome sequence of *B. amyloliquefaciens* EG025 has been deposited in the National Center for Biotechnology Information (NCBI) genome database under the accession number CP187241 (BioProject, PRJNA1247436; BioSample, SAMN47812635).

## Results

### Gliadin Degradation Activity of *B. amyloliquefaciens* EG025

**Fig. 1:**
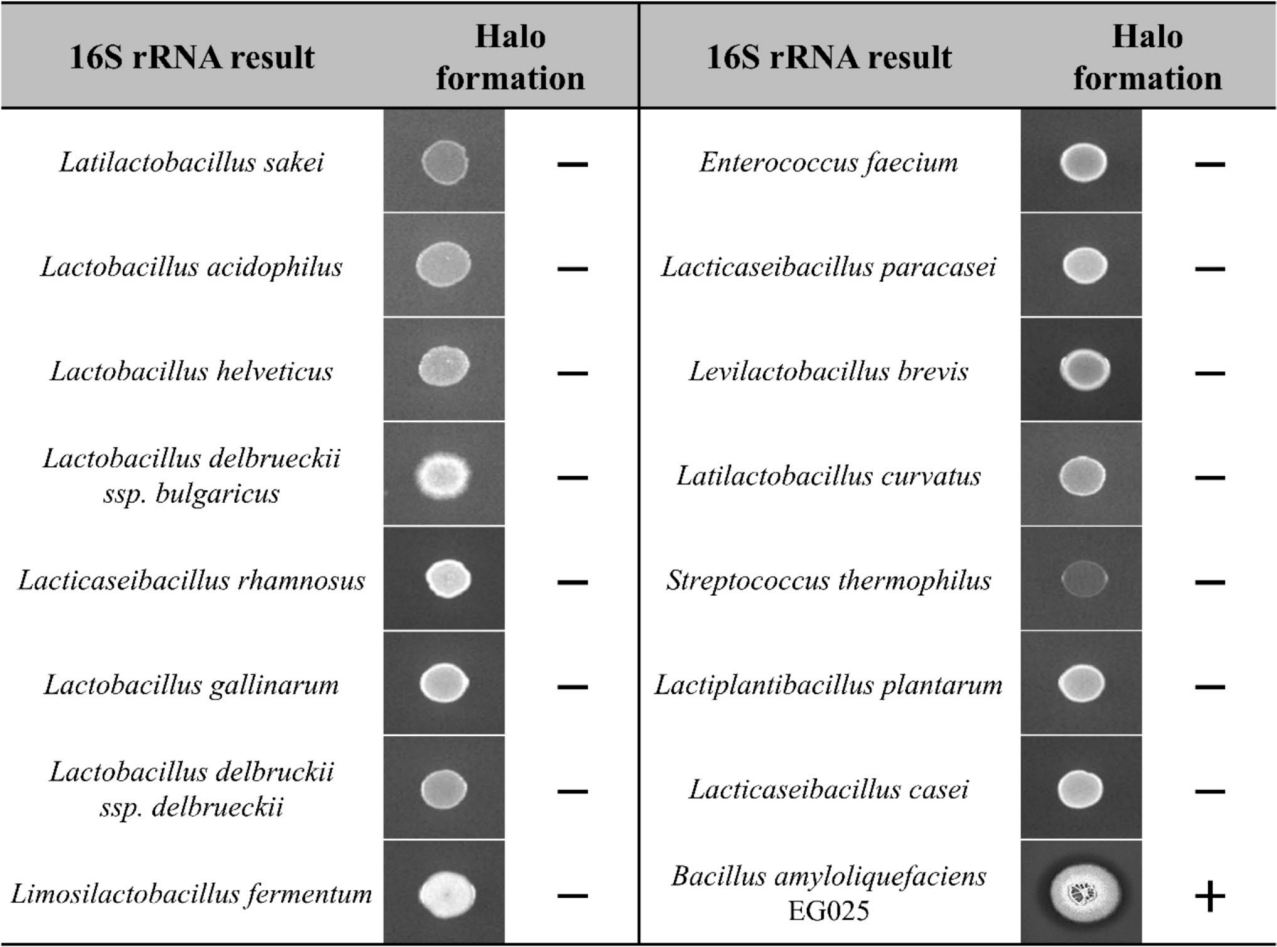
Identification of probiotic candidates based on colony morphology and halo formation. Among the 34 isolates obtained from 23 traditional fermented foods, only *Bacillus amyloliquefaciens* EG025 exhibited halo formation. Isolates not identified as lactic acid bacteria were excluded from the presented results. (Halo formation: −, no halo zone detected around colonies; +, halo zone detected around colonies)

To isolate probiotics capable of degrading gliadin, 23 traditional Korean fermented foods, including kimchi, jeotgal, and cheonggukjang, were used as the sources. Based on colony morphology, 42 strains were isolated, and subsequent 16S rRNA gene sequencing identified 34 potential probiotic strains. These included *lactobacilli* such as *Latilactobacillus sakei*, *Lactobacillus acidophilus*, and *Lacticaseibacillus rhamnosus, Streptococcus thermophilus*, as well as *Pediococcus pentosaceus*, *Weissella confusa*, *Weissella cibaria*, and *Bacillus amyloliquefaciens*. Among the proteins that constitute gluten, gliadin is known to be closely related to the pathogenesis of celiac disease [40]. Accordingly, these strains were screened for gliadin-degrading activity using TSA-gliadin plates. Notably, only one strain, *Bacillus amyloliquefaciens* EG025, exhibited a halo zone around its colonies, leading to its selection as a gliadin-degrading probiotic (Fig. 1).

### The Effect of pH on Gliadin Degradation

**Fig. 2:**
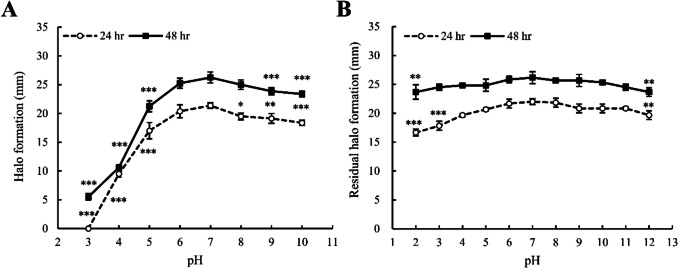
Effect of pH on the gliadin-degrading activity of *B. amyloliquefaciens* EG025.Changes in gliadin-degrading activity at different pH levels were measured based on the halo size. **(A)** Variation in gliadin-degrading activity as a function of pH. *B. amyloliquefaciens* EG025 exhibited the highest activity at neutral pH and maintained at least 86% of its maximum activity under alkaline conditions. **(B)** Residual activity following a 2-h exposure to various pH conditions, with subsequent recovery at neutral pH. After 2 h of exposure, activity was restored to up to 76% of the maximum. Statistical significance was determined by one-way ANOVA followed by Dunnett’s test, comparing each pH condition to the maximum activity (*p < 0.05, **p < 0.01, ***p < 0.001)

The direct effects of pH on gliadin degradation were evaluated by measuring halo formation on TSA-gliadin plates to determine if the gliadin-degrading activity of *B. amyloliquefaciens* EG025 remains robust in acidic environments. As a results, *B. amyloliquefaciens* EG025 consistently produced the largest halos within the neutral pH range (pH 6–8), irrespective of incubation time. Notably, the largest halo (25 mm) was observed at pH 7. Additionally, gliadin-degrading activity gradually decreased in more alkaline conditions (pH 9–10). Specifically, at pH 10, which exhibited the lowest observed activity, halos reached approximately 86% of the size observed at pH 7 after 24 h of incubation, increasing to 89% after 48 h. Conversely, halo sizes sharply decreased at acidic pH values below 5. At pH 5, halo formation was 81% of the maximum after 48 h but dropped significantly to only 40.5% at pH 4. Furthermore, at pH 3, no halo formation was observed after 24 h, and after an additional 24 h, halos were only 5.5 mm, representing merely 20% of the maximal halo size observed at neutral pH (Fig. 2A).

To further evaluate the recovery of gliadin-degrading activity after transient exposure to various pH environments, residual gliadin-degrading activities were measured after exposing *B. amyloliquefaciens* EG025 to different pH conditions (pH 2–12) for 2 h, followed by returning the environment to neutral pH. The strain again exhibited the highest residual activity at neutral pH (pH 6–8), with the largest halo averaging 26.2 mm at pH 7. In alkaline environments, residual activity declined slowly with increasing pH. Specifically, even at pH 12, where residual gliadin-degrading activity was decreased most significantly, halos remained at 89.4% of the maximum after 24 h, increasing slightly to 90.5% after 48 h. In contrast to direct gliadin-degrading activity, residual activity after exposure to acidic conditions significantly recovered. Residual activity at pH 5 to 2 after 24 h of incubation remained high at 93.9%, 89.4%, 81.1%, and 75.8%, and further increased after 48 h to 94.9%, 94.9%, 93.6%, and 90.4% respectively (Fig. 2B).

Dunnett’s statistical test comparing direct gliadin-degrading activity with residual activity revealed significant differences in gliadin-degrading activity across all non-neutral pH conditions. However, in residual activity comparisons, only halos produced at the extreme pH values (2 and 12) significantly differed from the maximum halo formation observed at pH 7.

### Acid and Bile Tolerance of *B. amyloliquefaciens* EG025

**Fig. 3:**
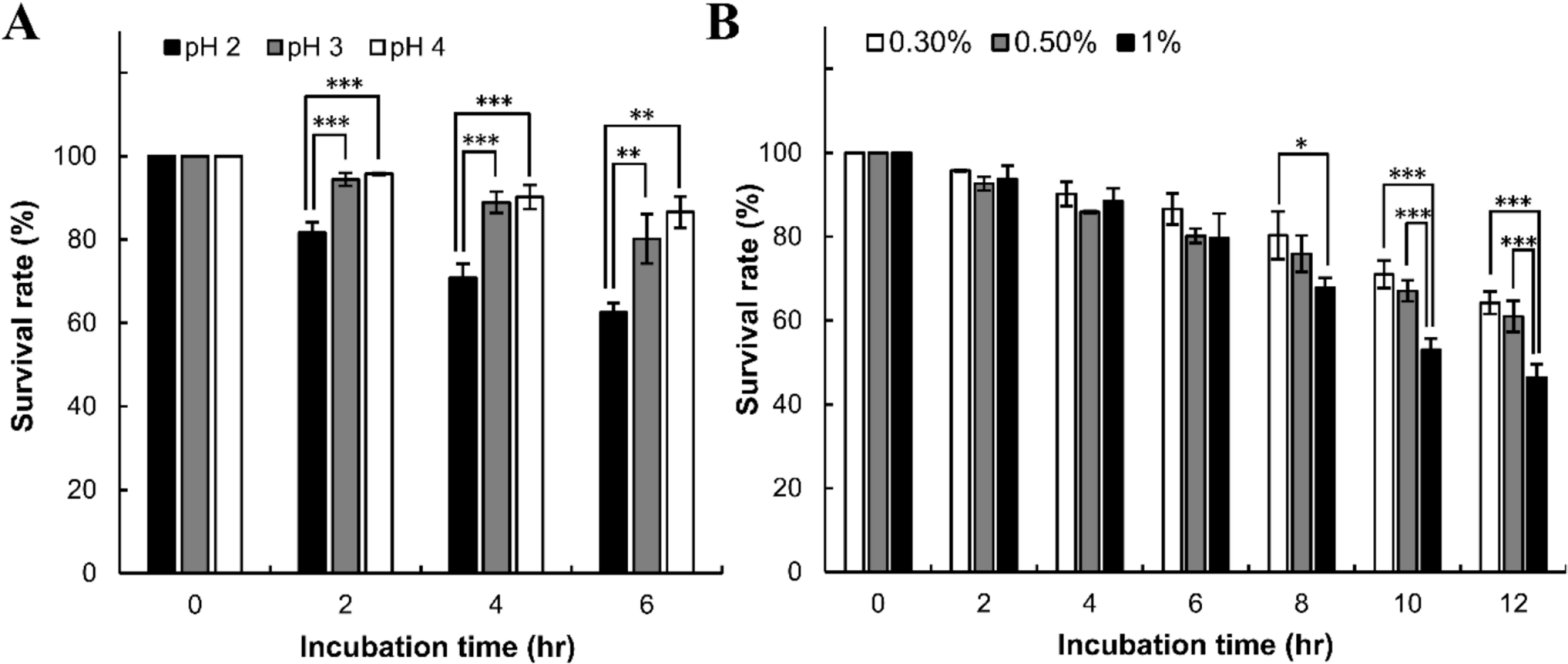
Results of acid and bile tolerance tests for assessing probiotic properties. **(A)** Acid tolerance test results for *B. amyloliquefaciens* EG025. It was observed that 62.5% of cells survived after 6 h of incubation at pH 2. In contrast, at pH 3 and pH 4, the survival rate decreased more gradually, with high survival rates of 80.2% and 86.6%, respectively, recorded after 6 hours. **(B)** Bile tolerance test results for *B. amyloliquefaciens* EG025. Under 1% bile conditions, cell survival was observed to decrease over time, with a survival rate of 46.4% recorded at 12 hours. In contrast, under 0.3% and 0.5% bile conditions, high survival rates of 64.2% and 61% were maintained, respectively. In both tests, a two-way ANOVA followed by Tukey-HSD post hoc analysis was performed to determine the conditions under which the decline in survival was most pronounced (*p < 0.05, **p < 0.01, ***p < 0.001)

To evaluate the acid tolerance of *B. amyloliquefaciens* EG025, its survival rate was assessed in a simulated gastric solution containing 3 mg/mL pepsin. The results indicated varying survival rates between pH 2 and 4, with significantly lower survival observed at pH 2 compared to pH 3 and 4, as confirmed by Tukey HSD statistical analysis. Specifically, at pH 2, *B. amyloliquefaciens* EG025 demonstrated a survival rate of 81.7% after 2 h of incubation, and despite a decreasing trend over time, maintained a relatively high survival rate of 62.5% even after 6 h. At pH 3, the survival rate gradually decreased to 94.4%, 88.9%, and 80.2% after incubation periods of 2, 4, and 6 h, respectively. Similarly, at pH 4, the strain showed survival rates of 95.8%, 90.2%, and 86.6% after 2, 4, and 6 h, respectively (Fig. 3A).

In the assessment of bile salt resistance, Tukey HSD statistical analysis revealed significant differences in survival rates between buffer solutions containing 1% bile salt and those containing 0.3% or 0.5%. Specifically, *B. amyloliquefaciens* EG025 exhibited a survival rate of 93.8% in a 1% bile salt solution after 2 h, subsequently decreasing to 88.5%, 79.7%, 67.8%, and 53% at incubation times of 4, 6, 8, and 10 h, respectively, reaching 46.4% after 12 h. At a lower bile salt concentration of 0.5%, survival rates were relatively higher, observed at 92.6%, 85.9%, 80.2%, 76%, 67.1%, and 61% for the corresponding incubation times. Further reduction of the bile salt concentration to 0.3% resulted in even higher survival rates of 95.8%, 90.2%, 86.6%, 80.3%, 71%, and 64.2% over the same incubation periods (Fig. 3B).

### Genomic Feature of *B. amyloliquefaciens* EG025

#### Genome Characteristics of *B. amyloliquefaciens* EG025

**Fig. 4:**
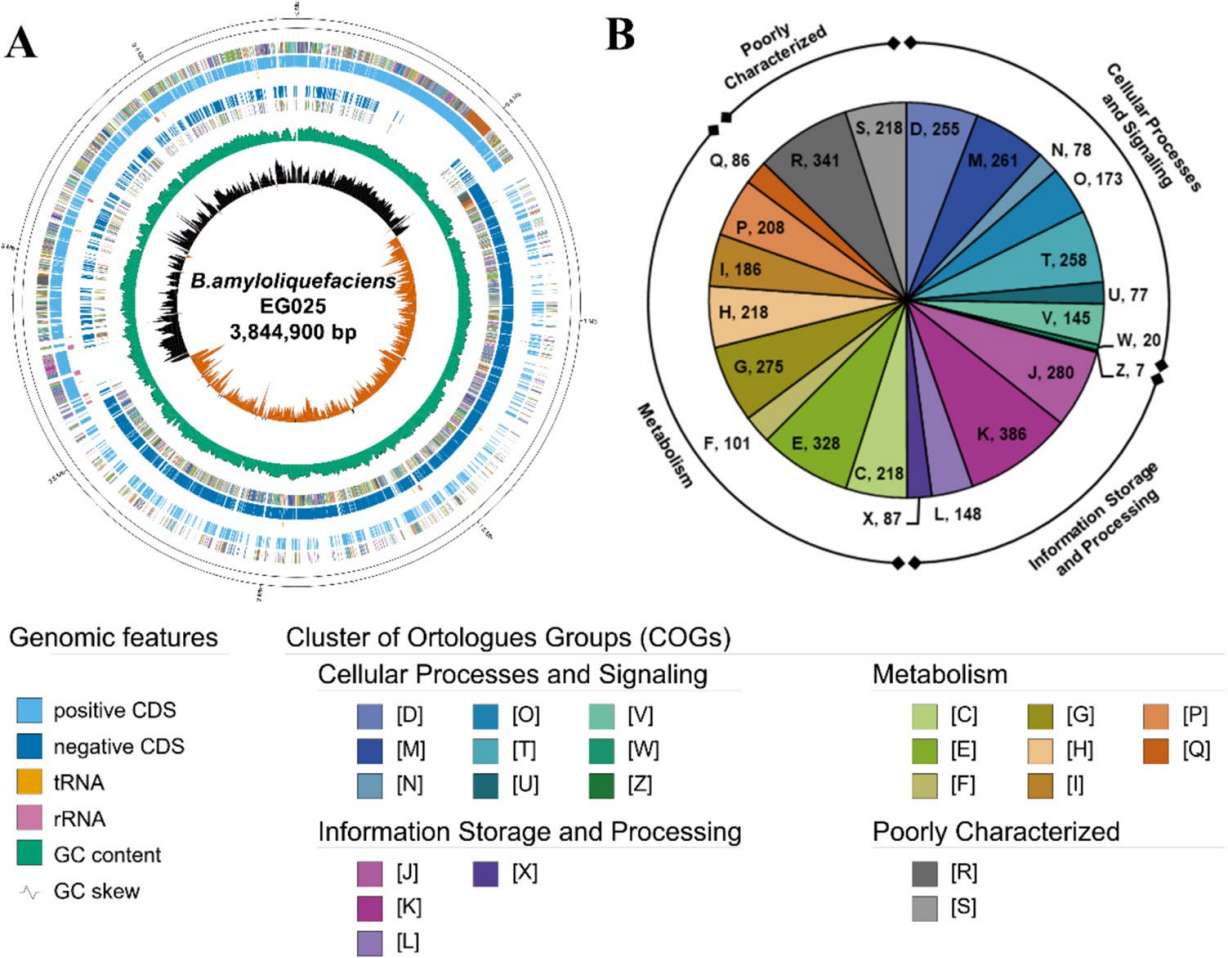
Genomic features of *B. amyloliquefaciens* EG025 resulted from the GenoVi program. **(A)** Circular map of *B. amyloliquefaciens* EG025, showing a genome size of 3,844,900 bp with a GC content of 46.04%. **(B)** Distribution of COG categories in the *B. amyloliquefaciens* EG025 genome. D, Cell cycle control, cell division, chromosome partitioning; M, Cell wall/membrane/envelope biogenesis; N, Cell motility; O, Posttranslational modification, protein turnover, chaperones; T, Signal transduction mechanisms; U, Intracellular trafficking, secretion, and vesicular transport; V, Defense mechanisms; W, Extracellular structures, Y, Nuclear structure; Z, Cytoskeleton; A, RNA processing and modification; B, Chromatin structure and dynamics; J, Translation, ribosomal structure and biogenesis; K, Transcription; L, Replication, recombination and repair; X, Mobilome: prophages, transposons; C, Energy production and conversion; E, Amino acid transport and metabolism; F, Nucleotide transport and metabolism; G, Carbohydrate transport and metabolism; H, Coenzyme transport and metabolism; I, Lipid transport and metabolism; P, Inorganic ion transport and metabolism; Q, Secondary metabolites biosynthesis, transport and catabolism; R, General function prediction only; S, Function unknown

In order to explore the prospective probiotic attributes of *B. amyloliquefaciens* EG025 at the genomic level, long read-based next-generation sequencing and whole genome assembly was carried out. As a result, a single contig was obtained and the assembly quality measured by the BUSCO value was 99.2%. Based on the whole genome sequence, a circular genome map was constructed and the structural features of *B. amyloliquefaciens* EG025 were examined. The genome was determined to be 3,844,900 bp in size with a GC content of 46.04% and comprised 3,850 coding sequences, 86 tRNA genes, 27 rRNA genes, one tmRNA, and 82 miscellaneous RNA genes (Fig. 4A).

The COG distribution of *B. amyloliquefaciens* EG025 revealed significant functional diversity associated with probiotic potential. Specifically, in the Cellular Processes and Signaling group, 255 genes were assigned to Category D (cell cycle control, cell division, and chromosome partitioning), 261 to Category M (cell wall/membrane/envelope biogenesis), 78 to Category N (cell motility), 173 to Category O (posttranslational modification, protein turnover, and chaperones), and 258 to Category T (signal transduction mechanisms). In addition, 77 genes were allocated to Category U (intracellular trafficking, secretion, and vesicular transport), 145 to Category V (defense mechanisms), 20 to Category W (extracellular structures), and 7 to Category Z (cytoskeleton), yielding a total of 1,274 genes for this group. In the Information Storage and Processing group, 280 genes were assigned to Category J (translation, ribosomal structure, and biogenesis), 386 to Category K (transcription), 148 to Category L (replication, recombination, and repair), and 87 to Category X (unassigned), totaling 901 genes. In the Metabolism group, 218 genes were classified under Category C (energy production and conversion), 328 under Category E (amino acid transport and metabolism), 101 under Category F (nucleotide transport and metabolism), 275 under Category G (carbohydrate transport and metabolism), 218 under Category H (coenzyme transport and metabolism), 186 under Category I (lipid transport and metabolism), 208 under Category P (inorganic ion transport and metabolism), and 86 under Category Q (secondary metabolites biosynthesis, transport, and catabolism), summing to 1,620 genes. The remaining 559 genes were allocated to poorly characterized categories (Fig. 4B).

**Fig. 5:**
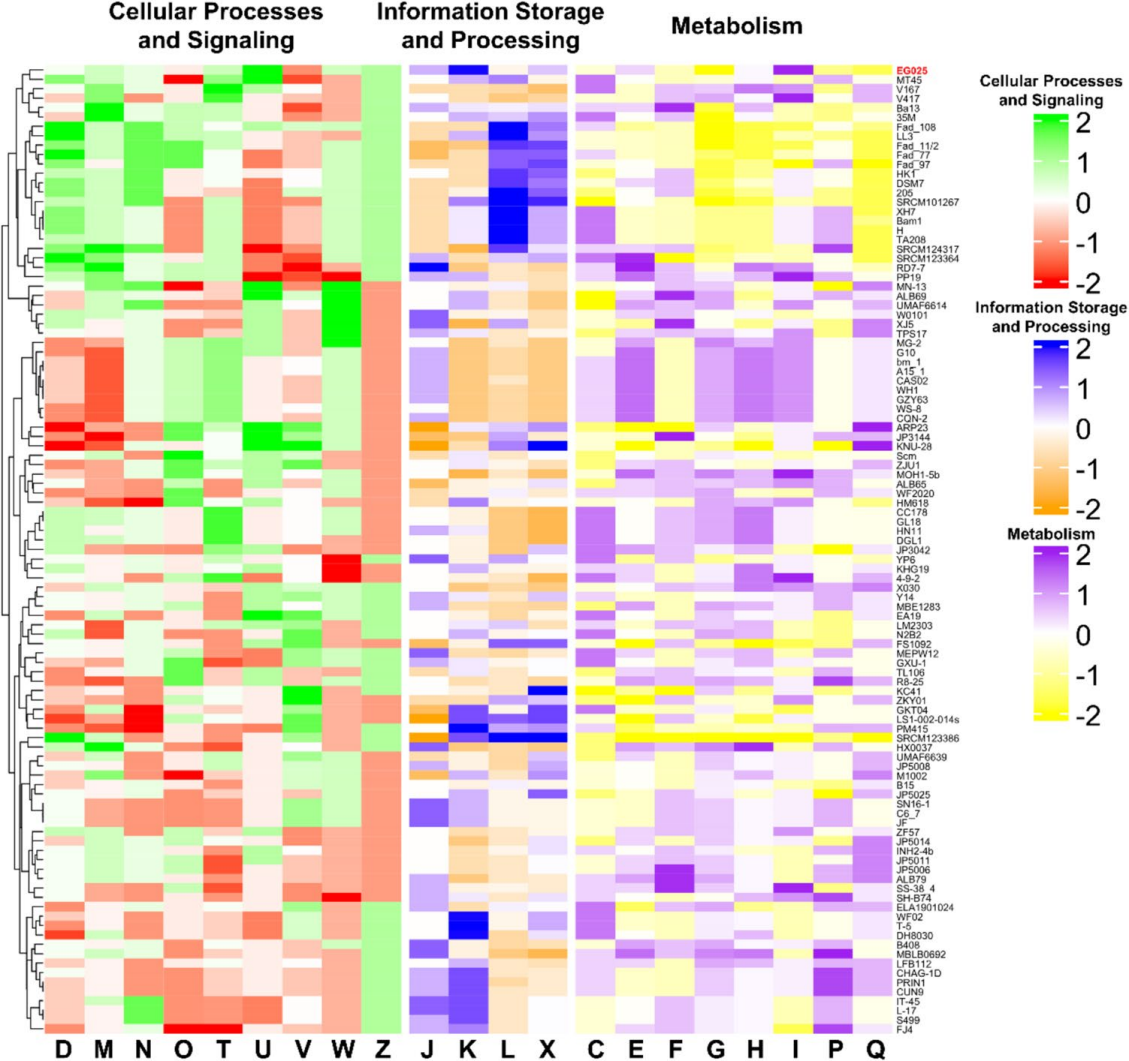
Comparative analysis result of COG distribution in *B. amyloliquefaciens* genomes.COG groups were divided into cellular processes and signaling (green and red), information storage and processing (blue and orange), and metabolism (purple and yellow). Relative frequency in each COG category was normalized by z-score. D, Cell cycle control, cell division, chromosome partitioning; M, Cell wall/membrane/envelope biogenesis; N, Cell motility; O, Posttranslational modification, protein turnover, chaperones; T, Signal transduction mechanisms; U, Intracellular trafficking, secretion, and vesicular transport; V, Defense mechanisms; W, Extracellular structures; Z, Cytoskeleton; J, Translation, ribosomal structure and biogenesis; K, Transcription; L, Replication, recombination and repair; X, Mobilome: prophages, transposons; C, Energy production and conversion; E, Amino acid transport and metabolism; F, Nucleotide transport and metabolism; G, Carbohydrate transport and metabolism; H, Coenzyme transport and metabolism; I, Lipid transport and metabolism; P, Inorganic ion transport and metabolism; Q, Secondary metabolites biosynthesis, transport and catabolism

To specifically assess the differences in COG distribution between EG025 and other previously reported *B. amyloliquefaciens* genomes, the relative frequencies of COG categories were compared across all strains. In the Cellular Processes and Signaling group, genes classified under categories M, T, U, W, and Z were more abundantly represented in EG025, with each showing a z-score of at least 0.5. Notably, the relative frequency of COG U was 1.8%, which was significantly higher than the average of 1.6% (SD = 0.090%), yielding a z-score of 2.039. In contrast, COG V in EG025 showed a relative frequency of 3.3% and a z-score of − 1.148 (average = 3.5%, SD = 0.178%), indicating a relatively lower distribution of genes in this category. Within the Information Storage and Processing group, COG J and COG K both had z-scores greater than 0.5. In particular, COG K showed a relative frequency of 8.9% and a z-score of 1.981 (mean = 8.457%, SD = 0.223), suggesting an enrichment of genes involved in transcription-related functions. In the Metabolism group, categories COG E and COG I also had z-scores above 0.5. COG I was especially notable, with a relative frequency of 4.4% and a z-score of 1.982 (mean = 4.083%, SD = 0.110), highlighting a higher abundance of genes related to lipid transport and metabolism in EG025. Conversely, COG categories F, G, P, and Q showed lower z-scores (≤ − 0.5), with COG G, associated with carbohydrate transport and metabolism, exhibiting a relative frequency of 6.3% and a z-score of − 2.136 (mean = 6.943%, SD = 0.301), indicating that this category was less represented in EG025 compared to other strains (Fig. 5).

#### Gliadin-Degrading and Probiotic Functional Genes in *B. amyloliquefaciens* EG025

tBLASTn analysis was performed using a custom database that integrated previously reported putative gliadin-degrading and probiotic functional genes from *Bacillus spp.* with *B. amyloliquefaciens* EG025 genome, thereby enabling an evaluation of the genomic gliadin-degrading probiotic potential of *B. amyloliquefaciens* EG025. As a result, two candidate genes, *npr* and *apr*, were identified to be involved in gliadin degradation. These genes encode a metalloprotease known as bacillolysin and a serine protease referred to as subtilisin BPN’, respectively. Both proteins were found to possess signal sequences required for extracellular secretion (Table 1). In addition, the tBLASTn analysis revealed the presence of genes involved in repair of damaged proteins, such as *dnaK, groES, groEL, grpE,* and *gsp13,* which are critical for stabilizing and refolding proteins under stress, were present. Among these, *dnaK*, *groES*, and *groEL* function as essential molecular chaperones that assist in preventing protein aggregation and maintaining cellular homeostasis under extreme conditions. Their roles extend beyond simple protein refolding, influencing bacterial survival in high-stress environments such as acid exposure and heat shock [41]. Additionally, genes facilitating the refolding or degradation of denatured proteins, including *clpC, clpP,* and *clpX*, were detected. In terms of DNA repair, *uvrA, uvrB, uvrC,* and *uvrD* were also identified. Furthermore, genes associated with bile tolerance were found. Notably, those with bile salt hydrolase activity (*bshA, bshB1, bshB2, bshC*) and other bile tolerance-related genes (*acrD, gpmI, cbh, lysP, nagB, pyrG, TC.BASS*) were present. For acid tolerance, genes encoding components of the proton-transporting ATPase complex (*atpA, atpB, atpC, atpD, atpF, atpG, atpH, atpI*), genes for the cellobiose PTS system (*chbA1, chbC2, chbC3*), and Na⁺-H⁺ antiporters (*nhaC1, nhaC2*) were detected (Table 2). In addition, universal stress proteins such as *uspA1* and *uspA2* were present, along with genes conferring resistance to various stresses. Genes associated with cold shock, including *cspB, cspC,* and *cspD*, were detected, while those involved in osmotic stress, such as the glycerol uptake facilitator (*GlpF*), components of the opu system (*opuAA, opuBB1, opuBB2, opuBB3, opuCA, opuCC, opuCD, opuD*), and *betB, gbsB,* were also identified. Additionally, genes implicated in oxidative stress defense, including *sodC, Bcp, sodA, gpx,* and *nsrR*, as well as genes contributing to virulence stress and carbon starvation resistance, were observed (Table 3). Moreover, genes associated with adhesion and aggregation were identified. Among these, genes involved in flagellar synthesis (members of the *flh*, *fli*, and *flg* families) and those related to extracellular protein processing and attachment, including *srtA, lspA, fbpA1, fbpA3, fbpB1, fbpB2,* and *fbpB3,* were detected, suggesting that these genes likely facilitate cell-surface interactions and biofilm formation (Table 4). Finally, genes involved in vitamin biosynthesis, which may contribute beneficially to host nutrition, were also detected. Genes responsible for thiamine production (*thiM, ppaX, dxs*), biotin synthesis (*bioB, bioY, accB, accC*), and riboflavin biosynthesis (*ribD, ribE, ribF, nusB*) were identified, in addition to those implicated in pyridoxine (*pdxK, pdxS, pdxT*) and folate synthesis (*folB, folE, folK, folP, panC, panD, hpt*). Furthermore, genes involved in lipoic acid biosynthesis (*lipA, lipM, ywfL*) were found (Table 5).

**Table 1:** Putative genes involved in gliadin degradation detected in *B. amyloliquefaciens* EG025 genome

Gene name	Predicted function	Identity (%)	Length (aa)	e-value	Coverage (%)	Signal peptide
*npr*	Bacillolysin [EC:3.4.24.28]	99.42	521	0	100	N’-MGLGKKLSVAVAASFMSLTISLPGVQA
*apr*	Subtilisin BPN’ [EC:3.4.21.62]	99.22	382	0	100	N’-MRGKKVWISLLFALALIFTMAFGSTSSAQA

**Table 2:** Putative genes involved in acid and bile salt tolerance detected in *B. amyloliquefaciens* EG025 genome

Gene name	Predicted function	Identity (%)	Length (aa)	e-value	Coverage (%)	Function
*dnaK*	molecular chaperone DnaK	99.67	612	0	100	Repair of damaged proteins
*groES*	chaperonin GroES	97.87	94	4.19E-62	100	
*groEL*	chaperonin GroEL	99.81	526	0	97	
*grpE*	molecular chaperone GrpE	100	150	8.11E-110	79	
*gsp13*	general stress protein 13	98.46	130	9.26E-81	100	
*clpC*	ATP-dependent Clp protease ATPbinding subunit ClpC	97.53	811	0	100	Refolding or degrading denatured proteins
*clpP*	ATP-dependent Clp endopeptidase proteolytic subunit ClpP	100	198	2.88E-148	100	
*clpX*	ATP-dependent Clp protease ATPbinding subunit ClpX	99.28	420	0	100	
*uvrA*	excinuclease ABC subunit A	99.37	957	0	100	DNA repair
*uvrB*	excinuclease ABC subunit B	99.38	644	0	97	
*uvrC*	excinuclease ABC subunit C	98.81	590	0	100	
*uvrD*	DNA helicase II/ATP-dependent DNA helicase PcrA [EC:3.6.4.12]	97.111	761	0	100	
*bshA*	l-malate glycosyltransferase [EC:2.4.1.-]	97.61	377	0	100	Deconjugation of bile salts
*bshB1*	N-acetylglucosamine malate deacetylase 1 [EC:3.5.1.-]	94.94	237	8.52E-173	100	
*bshB2*	N-acetylglucosamine malate deacetylase 2 [EC:3.5.1.-]	99.10	221	1.58E-167	100	
*bshC*	bacillithiol synthase	95.18	539	0	97	
*acrD1_*	arginine:ornithine antiporter/lysine permease	95.16	372	0	100	Bile tolerance
*acrD2_*	arginine:ornithine antiporter/lysine permease	95.11	368	0	100	
*gpmI*	2,3-bisphosphoglycerate-independent phosphoglycerate mutase [EC:5.4.2.12]	99.8	511	0	100	
*cbh*	choloylglycine hydrolase [EC:3.5.1.24]	96.95	328	0	90	
*lysP1*	arginine:ornithine antiporter/lysine permease	96.78	466	0	100	
*lysP2*	arginine:ornithine antiporter/lysine permease	97.67	472	0	100	
*lysP3*	arginine:ornithine antiporter/lysine permease	97.88	472	0	100	
*nagB*	glucosamine-6-phosphate deaminase [EC:3.5.99.6]	95.44	241	1.15E-177	100	
*pyrG*	CTP synthase [EC:6.3.4.2]	99.63	535	0	100	
*TC.BASS*	bile acid:Na + symporter, BASS family	87.94	315	0	97	
*TC.BASS*	bile acid:Na + symporter, BASS family	96.49	313	0	96	
*atpA*	F-type H+/Na + -transporting ATPase subunit alpha [EC:7.1.2.2 7.2.2.1]	99.80	502	0	100	Acid tolerance
*atpB*	F-type H + -transporting ATPase subunit a	99.18	244	0	100	
*atpC*	F-type H + -transporting ATPase subunit epsilon	98.49	132	3.59E-79	100	
*atpD*	F-type H+/Na + -transporting ATPase subunit beta [EC:7.1.2.2 7.2.2.1]	99.58	473	0	100	
*atpF*	F-type H + -transporting ATPase subunit b	99.41	170	3.37E-95	100	
*atpG*	F-type H + -transporting ATPase subunit gamma	99.30	287	0	100	
*atpH*	F-type H + -transporting ATPase subunit delta	100	181	1.04E-132	100	
*atpI*	ATP synthase protein I	98.43	127	2.14E-92	100	
*chbA1*	cellobiose PTS system EIIA component [EC:2.7.1.196 2.7.1.205]	98.08	104	1.64E-73	100	
*chbC2*	cellobiose PTS system EIIC component	97.12	452	0	100	
*chbC3*	cellobiose PTS system EIIC component	97.75	444	0	100	
*ldh*	l-lactate dehydrogenase [EC:1.1.1.27]	97.48	317	0	100	
*pgi*	glucose-6-phosphate isomerase [EC:5.3.1.9]	99.542	437	0	97	
*pyk*	pyruvate kinase [EC:2.7.1.40]	98.97	585	0	100	
*nhaC1*	Na+:H+ antiporter, NhaC family	96.66	419	0	100	
*nhaC2*	Na+:H+ antiporter, NhaC family	97.01	468	0	100

**Table 3:** Putative genes involved in stress response detected in *B. amyloliquefaciens* EG025 genome

Gene name	Predicted function	Identity (%)	Length (aa)	e-value	Coverage (%)	Condition
*uspA1*	TRAP-T-associated universal stress protein TeaD	96.62	148	3.76E-105	100	TRAP-T-associated universal stress protein
*uspA2*	TRAP-T-associated universal stress protein TeaD	97.59	166	2.01E-107	100	
*clpC*	ATP-dependent protease ATP-binding subunit ClpC	97.53	811	0	100	Damaged proteins
*clpE*	ATP-dependent protease ATP-binding subunit ClpE	96.56	698	0	100	
*clpP*	ATP-dependent Clp endopeptidase proteolytic subunit ClpP	100.00	198	1.81E-148	100	
*clpX*	ATP-dependent Clp protease ATP-binding subunit ClpX	99.29	420	0	100	
	Universal cold shock protein	97.10	69	4.25E-50	85	Cold shock proteins
*cspB*	Cold shock protein CspB	100.00	66	1.20E-45	100	
*cspC*	Cold shock protein CspC	98.51	67	2.30E-46	100	
*cspD*	Cold shock protein CspD	100.00	66	2.45E-45	100	
*GlpF*	Glycerol uptake facilitator and related aquaporins	96.73	275	0	100	Osmotic stress
*opuAA*	Glycine betaine transport ATP-binding protein	99.04	418	0	100	
*opuBB1*	Choline ABC transport system, permease protein	97.70	217	1.67E-124	100	
*opuBB2*	Choline ABC transport system, permease protein	95.39	217	8.01E-128	100	
*opuBB3*	Choline ABC transport system, permease protein	96.44	225	2.87E-136	100	
*opuCA*	Osmoprotectant ABC transporter ATP-binding protein OpuCA	98.68	379	0	100	
*opuCC*	Glycine betaine/carnitine/choline-binding protein	97.44	273	0	90	
*opuCD*	glycine betaine/carnitine/choline/choline sulfate ABC transporter permease	96.88	224	9.08E-147	100	
*opuD*	Glycine betaine transporter	96.68	512	0	100	
	Betaine/proline/choline family ABC transporter ATP-binding protein	96.59	381	0	100	
*betB*	Betaine-aldehyde dehydrogenase	99.18	490	0	100	
*gbsB*	Alcohol dehydrogenase GbsB (type III), essential for the utilization of choline (EC 1.1.1.1)	98.51	402	0	100	
*sodC*	Cu/Zn superoxide dismutase	93.88	196	2.52E-140	100	Oxidative stress
	Superoxide dismutase [Mn]	96.80	281	0	100	
*Bcp*	Peroxiredoxin	96.82	157	5.89E-116	100	
*sodA*	Superoxide dismutase SodA	99.50	201	1.04E-149	100	
*gpx*	Glutathione_peroxidase_(EC_1.11.1.9)	94.34	159	3.48E-116	99	
	Organic hydroperoxide resistance transcriptional regulator	96.45	141	1.01E-101	100	
	Organic hydroperoxide resistance transcriptional regulator	97.06	136	3.54E-86	100	
*nsrR*	Nitric oxide-sensing transcriptional repressor	97.22	144	5.52E-91	100	
*rasP*	Regulator of sigma-W protease RasP	97.62	420	0	100	Periplasmic Stress Response
*rasP*	Regulator of sigma-W protease RasP	93.10	420	0	100	
	NG,NG-dimethylarginine dimethylaminohydrolase 1 (EC 3.5.3.18)	92.66	286	0	100	Dimethylarginine metabolism
*yjbI*	Hemoglobin-like protein	95.00	100	4.22E-72	76	Bacterial hemoglobins
*hfq*	RNA chaperone Hfq	100.00	73	1.54E-51	100	virulence stress
*cstA*	Carbon starvation induced membrane protein	99.33	598	0	100	Carbon starvation
*cstA*	Carbon starvation induced membrane protein	98.83	598	0	100

**Table 4:** Putative genes involved in adhesion and aggregation detected in *B. amyloliquefaciens* EG025 genome

Gene name	Predicted function	Identity (%)	Length (aa)	e-value	Coverage (%)
*srtA*	sortase A [EC:3.4.22.70]	90.78	217	3.30E-149	100
*fbpA1*	iron (III) transport system substrate-binding protein	98.15	54	2.21E-36	95
*fbpA3*	iron (III) transport system substrate-binding protein	95.00	60	2.19E-40	100
*fbpB1*	iron (III) transport system permease protein	93.75	48	1.34E-29	100
*fbpB2*	iron (III) transport system permease protein	97.56	41	6.39E-25	100
*fbpB3*	iron (III) transport system permease protein	89.83	59	1.35E-25	97
*lspA*	signal peptidase II [EC:3.4.23.36]	98.57	140	1.61E-87	92
*eno*	enolase [EC:4.2.1.11]	100.00	430	0	100
*mtnW*	2,3-diketo-5-methylthiopentyl-1-phosphate enolase [EC:5.3.2.5]	97.77	404	0	100
*gapN*	glyceraldehyde-3-phosphate dehydrogenase (NADP+) [EC:1.2.1.9]	98.53	340	0	100
*gapN*	glyceraldehyde-3-phosphate dehydrogenase (NADP+) [EC:1.2.1.9]	100.00	335	0	100
*flhA*	flagellar biosynthesis protein FlhA	99.26	677	0	100
*flhB*	flagellar biosynthesis protein FlhB	98.61	359	0	99
*flhF*	flagellar biosynthesis protein FlhF	94.23	364	0	100
*fliC, hag*	flagellin	94.48	308	0	94
*fliD*	flagellar hook-associated protein 2	95.06	506	0	100
*fliE*	flagellar hook-basal body complex protein FliE	99.06	106	1.82E-57	100
*fliF*	flagellar M-ring protein FliF	96.64	536	0	100
*fliG*	flagellar motor switch protein FliG	99.70	338	0	100
*fliH*	flagellar assembly protein FliH	93.63	251	1.21E-145	100
*fliI*	flagellum-specific ATP synthase [EC:7.4.2.8]	98.86	438	0	100
*fliM*	flagellar motor switch protein FliM	99.10	332	0	100
*fliP*	flagellar biosynthesis protein FliP	98.19	221	2.02E-139	100
*fliQ*	flagellar biosynthesis protein FliQ	97.75	89	1.03E-48	100
*fliR*	flagellar biosynthesis protein FliR	98.46	259	3.51E-176	100
*fliS*	flagellar secretion chaperone FliS	96.24	133	1.18E-94	100
*flgB*	flagellar basal-body rod protein FlgB	96.12	129	6.35E-91	100
*flgC*	flagellar basal-body rod protein FlgC	96.00	150	1.08E-106	100
*flgD*	flagellar basal-body rod modification protein FlgD	92.47	146	3.16E-89	100
*flgE*	flagellar hook protein FlgE	72.35	264	4.56E-135	100
*flgG*	flagellar basal-body rod protein FlgG	98.06	258	0	100
*flgK*	flagellar hook-associated protein 1	98.81	505	0	100
*flgL*	flagellar hook-associated protein 3 FlgL	99.34	305	0	100

**Table 5:** Putative genes involved in vitamin biosynthesis detected in *B. amyloliquefaciens* EG025 genome

Gene name	Predicted function	Identity (%)	Length (aa)	e-value	Coverage (%)	Vitamin
*thiM*	hydroxyethylthiazole kinase	94.49	272	4.30E-168	100	Thiamine
*ppaX*	pyrophosphatase PpaX	98.15	216	1.59E-147	100	
	thiamine diphosphokinase	94.39	214	1.94E-154	100	
	GTP pyrophosphokinase	99.52	210	4.69E-158	100	
*dxs*	1-deoxy-D-xylulose-5-phosphate synthase	99.05	633	0	100	
*bioB*	biotin synthase BioB	98.23	339	0	100	Biotin
*BioY*	biotin transporter	94.60	185	1.22E-66	100	
*BioY*	biotin transporter	96.45	197	2.22E-99	100	
*accB*	acetyl-CoA carboxylase biotin carboxyl carrier protein	98.11	159	1.70E-90	100	
*accC*	acetyl-CoA carboxylase biotin carboxylase subunit	99.78	450	0	100	
	bifunctional biotin--[acetyl-CoA-carboxylase] synthetase/biotin operon repressor	97.85	325	0	100	
*ribD*	bifunctional diaminohydroxyphosphoribosylaminopyrimidine deaminase/5-amino-6-(5-phosphoribosylamino)uracil reductase RibD	95.15	371	0	100	Riboflavin
*ribE*	riboflavin synthase	98.14	215	2.20E-157	100	
*ribF*	bifunctional riboflavin kinase/FAD synthetase	95.21	313	0	100	
	diacylglycerol kinase family protein	96.77	124	5.30E-74	98	
	diacylglycerol kinase	98.35	303	0	100	
*nusB*	transcription antitermination factor	98.46	130	3.70E-95	100	
*pdxK*	pyridoxine pyridoxal and pyridoxamine kinase	99.63	270	0	100	Pyridoxine
*pdxS*	Pyridoxal biosynthesis lyase	99.66	294	0	100	
*pdxT*	glutamine amidotransferase	100.00	196	7.57E-148	100	
	5-formyltetrahydrofolate cyclo-ligase	88.04	184	2.09E-115	100	Folate
*folB*	dihydroneopterin aldolase	97.50	120	1.95E-88	100	
*folE*	GTP cyclohydrolase I	100.00	190	4.18E-143	100	
*folK*	2-amino-4-hydroxy-6-hydroxymethyldihydropteridine diphosphokinase	96.41	167	6.81E-124	100	
*folP*	dihydropteroate synthase	94.74	285	0	100	
	thymidylate synthase	99.16	238	1.22E-170	93	
*panC*	pantoate--beta-alanine ligase	96.15	286	0	100	
*panD*	aspartate 1-decarboxylase	97.64	127	3.80E-92	100	
*hpt*	hypoxanthine phosphoribosyltransferase	98.88	179	1.08E-128	100	
*lipA*	lipoyl synthase (lipoic acid synthetase)	100.00	298	0	100	Lipoic acid
*lipM*	putative lipoate protein ligase	99.64	278	0	100	
*ywfL*	Octanoyl-[GcvH]:protein N-octanoyltransferase	99.30	287	0	100	

#### Functional Metabolite Biosynthetic Gene Cluster in *B. amyloliquefaciens* EG025

**Fig. 6:**
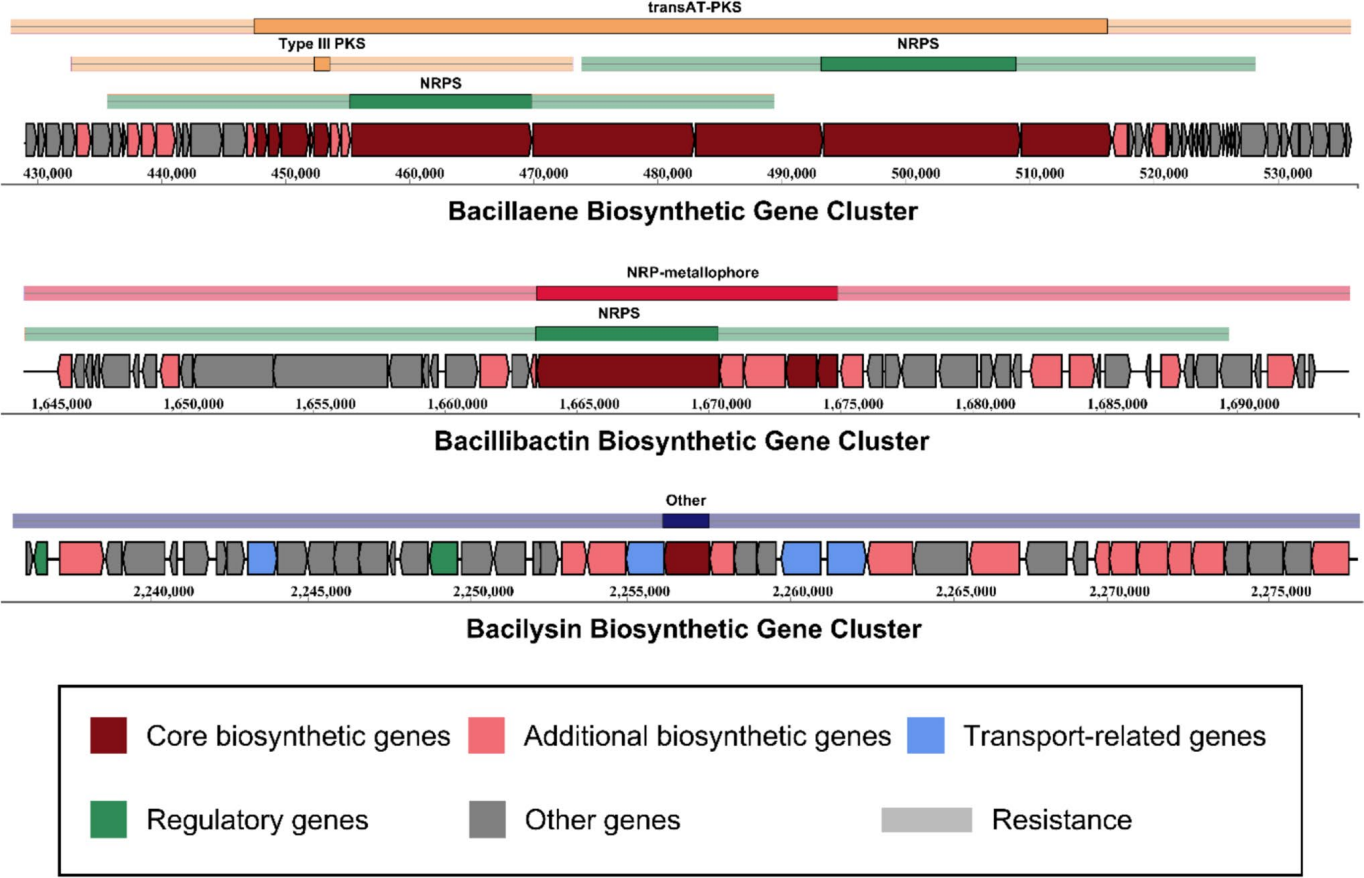
Representative biosynthetic gene clusters for secondary metabolites in the genome of *B. amyloliquefaciens* EG025. Among the 12 biosynthetic gene clusters identified in the genome of *B. amyloliquefaciens* EG025, three clusters showed 100% similarity to previously characterized gene clusters. These clusters were associated with the biosynthesis of bacillaene (upper panel), bacillibactin (middle panel), and bacilysin (lower panel), respectively

AntiSMASH analysis was performed to investigate the secondary metabolite biosynthetic gene clusters (BGC) present in the genome of *B. amyloliquefaciens* EG025, which may contribute to host health by synthesizing bioactive compounds with beneficial effects. The results revealed that the genome of EG025 harbors 12 distinct BGCs encoding secondary metabolites, including clusters for polyketide synthase (PKS), terpene, non‐ribosomal peptide synthetase (NRPS), and lanthipeptide. Among these, five BGCs displayed at least 80% similarity to previously reported biosynthetic gene clusters, and three clusters exhibited 100% similarity to known clusters (Supplementary Data, Figure S1). Notably, the BGC for bacillaene was located at positions 445,326–517,764 in the EG025 genome. This biosynthetic gene cluster was confirmed to comprise 9 core biosynthetic genes (*baeC*, *baeD*, *baeE*, Malonyl CoA‐acyl carrier protein transacylase; *baeG*, hydroxymethylglutaryl‐CoA synthase; *baeJ*, *baeN*, Condensation domain‐containing protein; *baeL*, short‐chain dehydrogenase/reductase SDR; *baeM*, *baeR*, Beta‐ketoacyl synthase) and 5 additional biosynthetic genes (*baeB*, hydroxyacylglutathione hydrolase; *acpK*, acyl carrier protein; *baeH*, *baeI*, enoyl‐CoA‐hydratase; *baeS*, putative cytochrome P450 107K1). Similarly, the BGC involved in bacillibactin biosynthesis was identified at positions 1,662,650–1,675,619. This cluster comprised 3 core biosynthetic genes (*dhbA*, short‐chain dehydrogenase/reductase SDR; *dhbC*, isochorismate synthase; *dhbF*, condensation domain‐containing protein) and 4 additional biosynthetic genes (*besA*, bacillibactin trilactone hydrolase; *dhbB*, isochorismatase; *dhbE*, AMP‐dependent synthetase and ligase; *mbtH*, mbtH‐like protein). Finally, the bacilysin BGC showed 100% similarity to a known cluster and was located at positions 2,253,753–2,261,050 in the EG025 genome. This cluster was composed of 1 core biosynthetic gene (*bacD*, argininosuccinate lyase/adenylosuccinate lyase), 2 additional biosynthetic genes (*bacC*, short‐chain dehydrogenase/reductase SDR; *ywfG*, aminotransferase), 2 transport‐related genes (*bacE*, *ywfA*, major facilitator transporter), and 2 other genes (*bacA*, prephenate dehydratase; *bacB*, isomerase) (Fig. 6).

### Safety Assessment for *B. amyloliquefaciens* EG025

#### Hemolytic Activity of *B. amyloliquefaciens* EG025

**Fig. 7:**
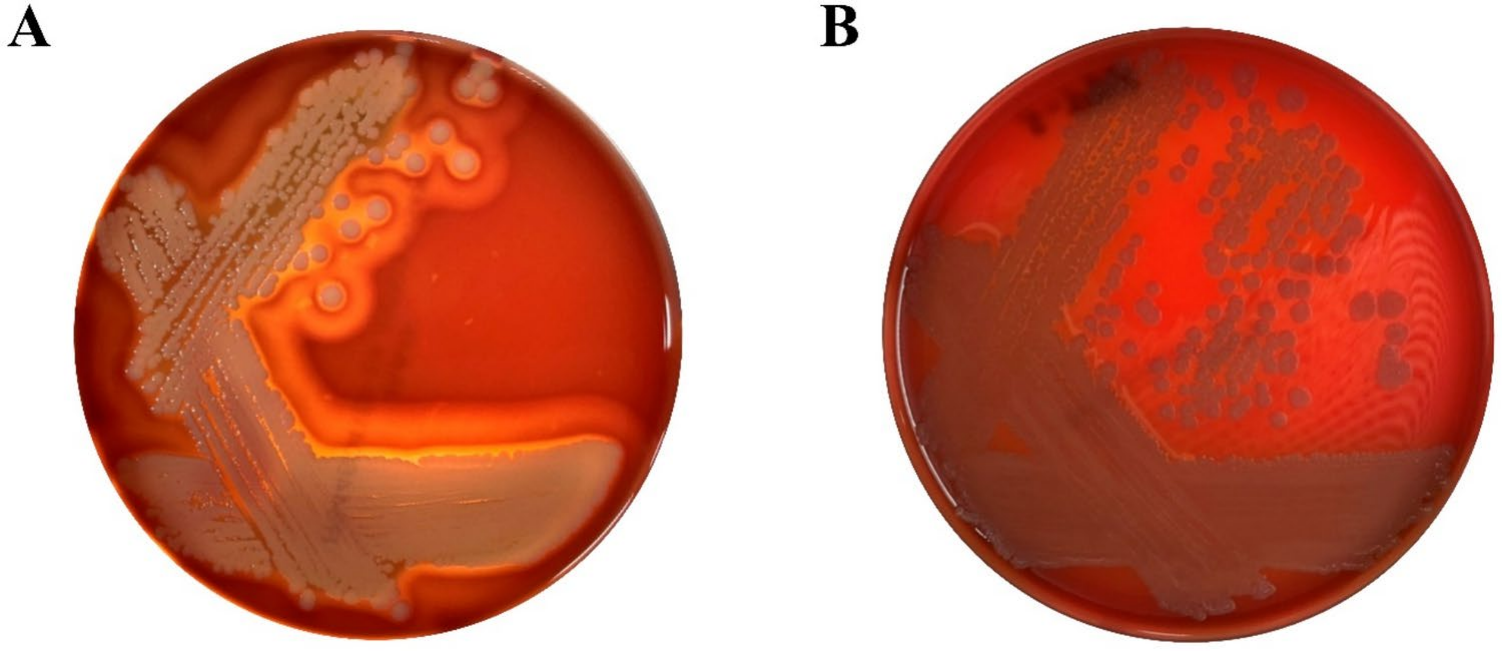
Hemolytic activity test result for safety assessment of *B. amyloliquefaciens* EG025. **(A)** *Staphylococcus aureus* produced a prominent clear zone surrounding its colonies, indicative of complete lysis (β-hemolysis). **(B)** In contrast, *B. amyloliquefaciens* EG025 exhibited no changes around the colonies, suggesting non-hemolytic activity (γ-hemolysis)

To examine whether *B. amyloliquefaciens* EG025 possesses hemolytic properties, the strain was cultured on sheep blood agar plates, with *S. aureus* included as a positive control for comparison [42]. Upon incubation, colonies of *B. amyloliquefaciens* EG025 displayed neither clear zones nor any visible alterations in the appearance of surrounding blood agar. Conversely, distinct clear zones indicating β-hemolysis were prominently formed around the colonies of *S. aureus* (Fig. 7).

#### Phenotypic Antibiotic Resistance of *B. amyloliquefaciens* EG025

The phenotypic antibiotic resistance pattern of *Bacillus amyloliquefaciens* EG025 was investigated using the Kirby-Bauer disk diffusion method against a set of 16 antibiotics from various classes commonly prescribed in clinical practice. The results indicated that *B. amyloliquefaciens* EG025 exhibited resistance only to aztreonam, a monobactam antibiotic. In contrast, the strain showed no resistance to penicillin-class antibiotics such as amoxicillin, amoxicillin-clavulanate, and amoxicillin-sulbactam, nor did it show resistance to cephem-class antibiotics including cefaclor, cephradine, and cefpodoxime, and it was also susceptible to the carbapenem antibiotic meropenem. Additionally, *B. amyloliquefaciens* EG025 exhibited no resistance to macrolide antibiotics such as clarithromycin and roxithromycin, to quinolone antibiotics such as ciprofloxacin and ofloxacin, to tetracycline-class antibiotics such as doxycycline and minocycline, to the aminoglycoside antibiotic ribostamycin, or to the rifamycin antibiotic rifamycin (Table 6A).

**Table 6:** Antibiotic resistance result of *B. amyloliquefaciens* EG025 for safety assessment. (A) Phenotypic antibiotic resistance result measured by Kirby-Bauer disc diffusion test using frequently prescribed antibiotics. (B) Comparison of detected gene counts between *B. amyloliquefaciens *EG025, LFB112, and TL106 using ABRicate across various databases

Category	Antibiotic	Concentration (µg)	Clear zone (mm)	Resistance
Penicillin	Amoxicillin-Clavulanate	20 – 10	25.5	**S**
	Amoxicillin	25	24	**S**
	Amoxicillin-Sulbactam	25 – 10	26.5	**S**
Cephem	Cefaclor	30	44.5	**S**
	Cephradine	30	39.5	**S**
	Cefpodoxime	10	27.5	**S**
Carbapenem	Meropenem	10	31	**S**
Monobactam	Aztreonam	30	9.5	**R**
Macrolide	Clarithromycin	15	28.5	**S**
	Roxithromycin	30	42.5	**S**
Quinolone	Ciprofloxacin	5	31	**S**
	Ofloxacin	5	30	**S**
Tetracycline	Doxycycline	30	34.5	**S**
	Minocycline	30	30.5	**S**
Aminoglycoside	Ribostamycin	50	29	**S**
Rifamycin	Rifamycin	10	35.5	**S**
			**Probiotic strain**	
Category	**Database**	***B. amyloliquefaciens*EG025**	***B. amyloliquefaciens*LFB112**	***B. amyloliquefaciens*TL106**
Antibiotic resistance gene	ARG-ANNOT	2	2	2
	CARD	2	1	1
	MEGARES	3	3	3
	NCBI	3	4	4
	PlasmidFinder	**0**	**0**	2
	ResFinder	1	2	2
Virulence factor	EcOH	**0**	**0**	**0**
	ecoli_vf	**0**	**0**	**0**
	VFDB	**0**	**0**	**0**

#### Antibiotic Resistance Genes and Virulence Factors in *B. amyloliquefaciens* EG025

To investigate the presence of antibiotic resistance genes and virulence factors in *B. amyloliquefaciens* EG025, a genome-wide screening was carried out using the ABRicate software. As a result, one to three antibiotic resistance genes were detected across the antibiotic resistance gene databases, while no sequences were identified in the PlasmidFinder database or the three virulence factor databases. For comparison, two known probiotic strains, *B. amyloliquefaciens* LFB112 and TL106, were analyzed under the same conditions. In the genome of LFB112, one to four antibiotic resistance genes were detected across the databases, and a similar result was observed for TL106, with the exception of the PlasmidFinder database. Notably, in both strains, no virulence factors were detected, consistent with the findings for EG025 (Table 6B). Subsequently, the specific genes identified in each database were examined. Three genes were commonly found in all three strains: *cfrB*, which encodes 23S rRNA (adenine(2503)-C(8))-methyltransferase (the same protein is annotated as *clbA* in the CARD, MEGARES, and NCBI databases); *satA*, which encodes streptothricin N-acetyltransferase; and *rphB*, which encodes rifamycin-inactivating phosphotransferase (registered as *rphC* in the NCBI database, although it encodes the same protein). In addition to these common genes, *tetL*, encoding a tetracycline efflux MFS transporter, was found in the genome of LFB112 but not in EG025. In TL106, both *tetL* and partial sequences matching *Salmonella enterica* subsp. *enterica* serovar Dublin plasmid pSD853_7.9 were detected (Supplementary Data, Table S1).

### Phylogenetic Relationship of *B. amyloliquefaciens* EG025

**Fig. 8:**
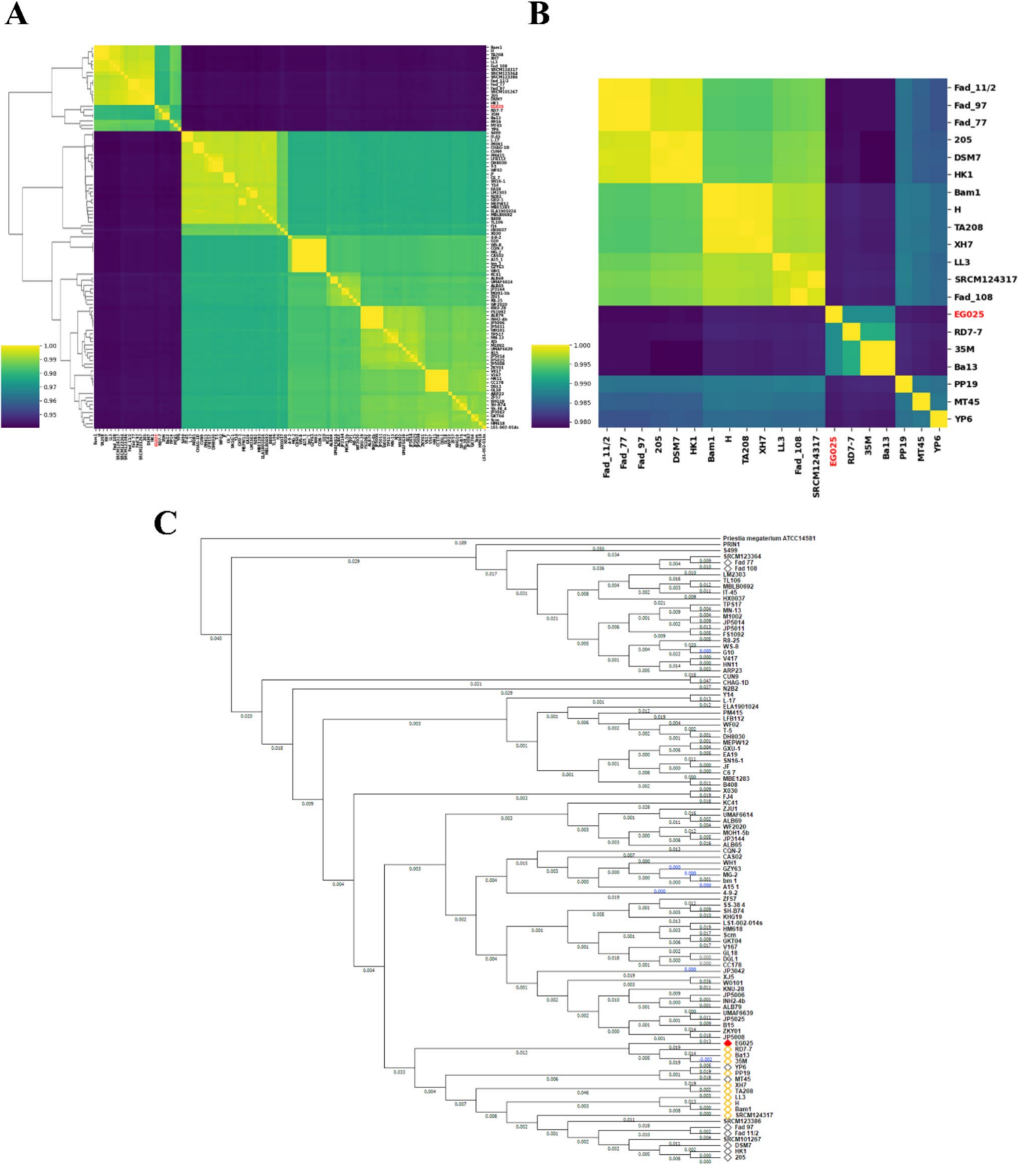
Phylogenetic analysis results for assessing genetic distances among *B. amyloliquefaciens* strains. A total of 103 *B. amyloliquefaciens* genomes were used to perform the phylogenetic analyses by both methods. **(A)** A cluster heatmap was generated based on ANI values calculated using PyANI, and several similar groups among the strains were observed. **(B)** A cluster heatmap of 19 strains determined to be genetically close to EG025. Among these, EG025 exhibited a clearly distinct position relative to other strains. **(C)** A phylogenetic tree was constructed using CVtree. Among the 19 strains identified as genetically close to EG025 by PyANI, all except Fad77 and Fad108 were also found to be closely related to EG025 in the tree. In contrast, SRCM101267 and SRCM123386, which were determined by PyANI to be relatively distant from EG025, were shown to be genetically close to EG025 in the CVtree results

To elucidate the genetic distances between *B. amyloliquefaciens* EG025 and other strains, full-genome based phylogenetic analyses were conducted on 103 *B. amyloliquefaciens* strains using PyANI and CVtree. The PyANI-based analysis revealed that the 103 strains clustered into several groups according to their ANI values, with strain RD7-7 exhibiting the highest ANI (98.877%) and strain CUN9 the lowest (94.046%) (Fig. 8A). Furthermore, strains RD7-7 (98.877%), 35M (98.874%), Ba13 (98.869), Bam1 (98.010%), H (98.009%), PP19 (98.002%), TA208 (97.995%), XH7 (97.987%), SRCM124317 (97.982%), and LL3 (97.981%) exhibited higher ANI values relative to *B. amyloliquefaciens* EG025, confirming their relatively close genetic relationship (Fig. 8B). Similarly, CVtree-based phylogenetic analysis revealed that *B. amyloliquefaciens* strains were classified into several distinct subgroups reflecting their genomic differences. Among the 103 genomes analyzed, 17 strains including *B. amyloliquefaciens* RD7-7, Ba13, 35 M, YP6, PP19, MT45, XH7, TA208, LL3, H, Bam1, SRCM124317, Fad 97, Fad 11/2, DSM7, HK1, and 205, which were previously identified via PyANI as closely related to EG025, formed one subgroup. However, in the CVtree results, strains Fad 77 (97.849% ANI) and Fad 108 (97.966% ANI), which exhibited similar ANI values in the PyANI analysis, were found to be relatively distant from EG025. Conversely, strains SRCM 123386 (97.843% ANI) and SRCM101267 (97.844% ANI), which were observed to be more distantly related to EG025 in the PyANI analysis than Fad 77 and Fad 108, were instead incorporated into the same subgroup (Fig. 8C).

## Discussion

This study aimed to identify potential probiotic bacteria for celiac disease management by discovering a bacterium capable of degrading gliadin with sufficient activity in the human gastrointestinal tract and by characterizing its probiotic properties and safety through both experimental and genomic approaches. To this end, *B. amyloliquefaciens* EG025 was identified as the sole strain among 28 isolates from 23 traditional Korean fermented foods to exhibit clear gliadin‑degrading activity by forming clear halos around the colonies. These results suggest that gliadin-degrading activity is a unique probiotic trait that is not commonly observed among the strains tested [43].

In mammals, the low pH of gastric juice often inactivates exogenously administered enzymes or probiotics [44, 45]. To evaluate whether the gliadin-degrading capability of *B. amyloliquefaciens* EG025 remains sufficiently active in the mammalian gastrointestinal tract, the effect of pH on its gliadin-degrading activity was investigated. In this result, *B. amyloliquefaciens* EG025 exhibited the highest gliadin-degrading activity in the neutral pH range (pH 6–8). Although the Dunnett test confirmed that the differences were statistically significant, the strain maintained at least 86% of its maximal activity in the alkaline range (pH 9–10). However, as the pH decreased below 5, its gliadin-degrading activity declined sharply. These results indicate that while the gliadin-degrading activity of EG025 is well sustained across a broad range encompassing neutral and alkaline conditions, it decreases dramatically under acidic conditions. Nevertheless, according to Yamamura et al., different segments of the human digestive system maintain distinct pH levels. For instance, the stomach typically maintains a pH of 1.0–2.0, while the pH rises to 6.1–7.5 after food passes through the stomach [46]. This suggests that the restoration of gliadin-degrading activity following exposure to acid may be more important than the direct inhibitory effects of low pH. Indeed, after exposing *B. amyloliquefaciens* EG025 to pH 3 for 2 h and subsequently returning the pH to neutral, 93.6% of its activity was recovered, and even after exposure to pH 2, a recovery of 90.4% was observed. These findings suggest that the residual gliadin-degrading activity remains largely unaffected by pH after a 2-h exposure, and considering that digestible solids are typically emptied from the human stomach within 2–3 h after a meal, *B. amyloliquefaciens* EG025 is likely to retain sufficient gliadin-degrading activity in the small intestine and colon even after passage through the low-pH gastric environment [47].

Probiotics exert their effects by colonizing the human gut. Therefore, high survival in digestive fluids containing acid and bile is one of the essential characteristics required of a probiotic [7]. To evaluate whether *B. amyloliquefaciens* EG025 possesses sufficient resistance to acid and bile for probiotic use, its survival was measured in simulated gastric and bile solutions. The results showed that EG025 exhibited varying survival rates from pH 2 to 4, and subsequent Tukey-HSD analysis confirmed that survival at pH 2 was significantly lower than at pH 3–4. Nonetheless, after incubating at pH 2 for 2 h, the strain maintained a high survival rate of 81.7%, and after 6 h, which exceeds the typical residence time of food in the stomach, its viability remained above 62.5%. It is generally known that human gastric juice contains between 0.576 and 0.865 mg/mL of pepsin, a level that not only aids in protein digestion but also contributes to the destruction of ingested microorganisms [48, 49]. Accordingly, it is noteworthy that *B. amyloliquefaciens* EG025 maintains a high survival rate in a simulated gastric solution with a pepsin concentration three times higher than that found in vivo. Similarly, the evaluation of survival in a bile solution containing 0.3–1% bile salt revealed that *B. amyloliquefaciens* EG025 exhibited high survival. In the 1% bile solution, *B. amyloliquefaciens* EG025 showed an 80% survival rate after 6 h of incubation, and even when the incubation time was extended to 12 h, 46.4% of the cells remained viable. Given that the bile salt concentration in the human small intestine (from the duodenum to the ileum) is approximately 0.11 to 0.47% (2.43 to 10.34 mM), assuming an average molecular weight of 400–500 g/mol for bile salts such as taurocholate, glycocholate, and deoxycholate [50], the survival of *B. amyloliquefaciens* EG025 in bile solutions containing 0.3 or 0.5% bile salt, with survival rates of 64.2% and 61% after 12 h respectively, is still considered high. According to our previous study, *Lacticaseibacillus rhamnosus* GG (LGG), a commercially available probiotic strain with well-documented health benefits, exhibited a survival rate of 49% after 3 h at pH 2.5, whereas *B. amyloliquefaciens* EG025 showed greater acid tolerance, maintaining a higher survival rate of 62.5% even after incubation in simulated gastric fluid at pH 2 for 6 h. Similarly, in our prior investigation assessing bile tolerance, LGG demonstrated a survival rate of 30% after 6 h incubation in a 0.3% bile solution, whereas EG025 exhibited a significantly higher survival rate of 71% under identical conditions in this study [51]. These findings suggest that *B. amyloliquefaciens* EG025 is likely to exhibit sufficient survival during transit through the human digestive system and while colonizing the gut. Such high survival rates are commonly observed in strains belonging to the *Bacillus spp.* and are attributed both to their ability to form endospores in extreme conditions and to the higher proportion of saturated fatty acid chains relative to unsaturated ones in the phospholipid composition of their cell membranes [52, 53].

In order to explore the prospective probiotic attributes of *B. amyloliquefaciens* EG025 at the genomic level, long read-based next-generation sequencing and whole genome assembly was carried out. As a result, a single contig was obtained and the assembly quality measured by the BUSCO value was 99.2%. Given that a well-performed assembly is defined by a BUSCO value of at least 95%, the genome of *B. amyloliquefaciens* EG025 produced in this study has sufficient quality for further analyses [54]. The analysis of COG distribution is considered critical for the systematic understanding of major bacterial functions including metabolism, conservation, and information processing [55]. The comparative COG distribution analysis revealed distinctive functional profiles in *B. amyloliquefaciens* EG025, reflecting both its origin from fermentation and its probiotic potential. In cellular processes and signaling group, categories such as U (Intracellular trafficking, secretion, and vesicular transport) were significantly more abundant in EG025 (z-score = 2.039), suggesting enhanced secretion systems. These mechanisms are critical for exporting enzymes such as gliadin-degrading proteases and promoting cell–host interactions, aligning with its isolation from fermented food where enzyme secretion is essential for microbial growth. Conversely, a relative depletion in category V (Defense mechanisms) may indicate lower investment in stress defence, potentially reflecting adaptation to the nutrient-rich cheonggukjang environment [56]. In information storage and processing group, an enrichment in transcription-related genes (COG K; z-score = 1.981) suggests robust regulatory capacities. This aligns with studies showing that increased transcriptional machinery is linked to stress resilience in *Bacillus* strains during gut passage [57]. In metabolism group, elevated frequencies in amino acid (COG E; z-score = 0.531) and lipid metabolism (COG I; z-score = 1.982) pathways highlight EG025’s metabolic versatility. Notably, research on *B. amyloliquefaciens* AV5 in aquaculture demonstrated that enhanced lipid metabolism improves host growth and resilience [58]. A significant reduction in carbohydrate metabolism genes (COG G; z-score = –2.14) may reflect dietary adaptation that EG025 may rely less on simple sugars abundant in cheonggukjang and more on other nutrient sources.

According to the study by Lee et al., *B. amyloliquefaciens* subsp. *plantarum* exhibited substantial gluten-degrading activity even in the crude enzyme extract obtained after cell disruption, suggesting that the gliadin-degrading capability of *B. amyloliquefaciens* is more plausibly attributed to the action of specific enzymes rather than a specialized metabolic pathway [59]. In addition, several enzymes including neutrase, thermolysin, subtilisin, and bacillolysin derived from *Bacillus* spp. have been previously reported to effectively degrade gluten or gliadin [60–62]. Through this approach, candidate genes potentially involved in gliadin degradation were screened by first characterizing the known enzymatic properties and genetic information of such proteins. As a result, two candidate genes, *npr* and *apr*, were identified in the genome of strain EG025. These genes were found to encode a metalloprotease known as bacillolysin and a serine protease known as subtilisin BPN’, respectively. Previous studies have demonstrated that bacillolysin reduces dough rigidity by cleaving peptide bonds within the gluten network [62], while subtilisin effectively degrades gliadin. In particular, subtilisins are non-specific peptidases with affinity for hydrophobic amino acid residues in the substrate’s peptide chain, and they are capable of hydrolyzing peptide bonds formed by residues such as Ala, Leu, Ser, as well as Val, Tyr, Phe, Gln, and His [61]. Moreover, both proteins were predicted to possess N-terminal signal sequences required for extracellular secretion, further supporting the possibility that these enzymes are secreted into the extracellular space where they can act on gliadin.

For the detailed understanding of the functional gene profile underlying the probiotic potential of *B. amyloliquefaciens* EG025, comprehensive functional genes related to four major probiotic functions including acid and bile tolerance, adhesion and aggregation, stress response, and vitamin biosynthesis were analyzed. In the acid and bile tolerance category, several gene sets related to intracellular pH regulation, protein repair, and bile salt detoxification were identified, including molecular chaperones (*dnaK*, *groES*, *groEL*), general stress proteins (*gsp13*), and molecular chaperone GrpE (*grpE*). The presence of these genes suggests robust mechanisms for maintaining cellular integrity under acidic and bile-rich gastrointestinal conditions [63]. For adhesion and aggregation, gene groups involved in cell surface modification and extracellular polymer synthesis were identified, including those encoding sortase A (*srtA*), iron (III) transport system substrate-binding proteins (*fbpA1*, *fbpA3*), permease proteins (*fbpB1*, *fbpB2*, *fbpB3*), enolase (*eno*), and glyceraldehyde-3-phosphate dehydrogenase (*gapN*). The presence of these genes indicates that *B. amyloliquefaciens* EG025 is likely well-equipped for adherence to intestinal mucosa and biofilm formation [63]. Regarding stress response, the genome harbored genes associated with universal stress proteins (*uspA1*, *uspA2*), ATP-dependent proteases (*clpC*, *clpE*, *clpP*), glutathione peroxidase (*gpx*), and superoxide dismutase (*sodA*, *sodC*). These genes are crucial for managing oxidative stress, repairing damaged proteins, and enhancing overall resilience during gastrointestinal transit [64]. Finally, genes involved in the biosynthesis of essential vitamins, such as thiamine, biotin, riboflavin, pyridoxine, folate, and lipoic acid were detected. These genes highlight the strain's capability to potentially provide nutritional benefits through vitamin production [65]. From the antiSMASH results, the genome of EG025 harbored 12 distinct BGCs encoding secondary metabolites, including polyketide synthase (PKS), terpene, non-ribosomal peptide synthetase (NRPS), and lanthipeptide clusters. Among these, three clusters exhibited 100% identity to previously characterized BGCs involved in the biosynthesis of bacillaene, bacillibactin, and bacilysin. Bacillaene, a polyene antibiotic, inhibits various pathogenic bacteria, thereby enhancing colonization by beneficial microbes and improving host’s gut health by maintaining intestinal microbiota homeostasis and regulating immune response [66]. Bacillibactin, a siderophore, facilitates iron uptake in iron-limited environments, providing a competitive advantage by restricting pathogen growth and supporting survival in the gut [67]. Bacilysin, a dipeptide antibiotic, exhibits potent antimicrobial activity against Gram-positive bacteria and fungi, contributing to microbiota modulation and protecting the host against pathogens [68]. Overall, the whole-genome analysis demonstrates that *B. amyloliquefaciens* EG025 is enriched in COG categories related to secretion, regulation, metabolic adaptability, and stress resistance and reinforce its potential as a probiotic tailored by the fermentation environment. The analysis also shows that the strain harbors biosynthetic clusters for bioactive metabolites which enhance competitive fitness and host protection, underscoring its strong potential as an effective probiotic.

In assessing probiotic safety, it is critically important to verify the absence of hemolytic activity, transferable antibiotic resistance, and virulence factors [69]. In hemolytic activity evaluation result, colonies of *B. amyloliquefaciens* EG025 displayed neither clear zones nor any visible alterations in the appearance of surrounding blood agar and lacks hemolytic activity. In addition, the antibiotic resistance pattern of *B. amyloliquefaciens* EG025 indicated that *B. amyloliquefaciens* EG025 exhibited resistance only to aztreonam, which belongs to the monobactam antibiotic class. Conversely, susceptibility was observed against all other 15 antibiotics tested. The observed resistance against aztreonam is consistent with Clinical Laboratory Standards Institute (CLSI) and European Committee on Antimicrobial Susceptibility Testing (EUCAST), which advise the use of aztreonam susceptibility testing exclusively for Gram-negative bacteria [70, 71]. This recommendation is primarily based on narrow spectrum of aztreonam activity, specifically targeting Gram-negative bacteria due to its unique affinity to penicillin-binding protein 3 (PBP3), an enzyme predominantly found in Gram-negative organisms [72]. Consequently, the intrinsic resistance exhibited by Gram-positive bacteria such as *B. amyloliquefaciens* EG025 to aztreonam is expected and indicate the absence of significant antibiotic resistance or a safety concern for probiotic use. To investigate the presence of antibiotic resistance genes and virulence factors in the genome of *B. amyloliquefaciens* EG025, genome-wide screening was conducted against six antibiotic resistance gene databases and three virulence factor databases. As a result, the genomes of EG025 and the probiotic strains *B. amyloliquefaciens* LFB112 and TL106 were commonly found to contain three antibiotic resistance genes: *cfrB*, encoding 23S rRNA (adenine(2503)-C(8))-methyltransferase; *satA*, encoding streptothricin N-acetyltransferase; and *rphB*, encoding rifamycin-inactivating phosphotransferase. The *cfrB*, *satA*, and *rphB* genes have been primarily associated with resistance to lincosamides, macrolides, and streptogramins; streptothricins; and rifamycins, respectively. Interestingly, although the *rphB* gene identified in the EG025 genome exhibited identity and coverage values above the EFSA-recommended thresholds (minimum identity ≥ 80%, minimum coverage ≥ 70%) when compared with entries in antibiotic resistance gene databases, EG025 was phenotypically found to be susceptible to rifamycin in our antibiotic resistance test result. This observation is likely to be common among the three strains and may be explained by the absence of the rifampin-associated element (RAE). According to Spanogiannopoulou et al., proper expression of rifamycin inactivating phosphotransferase (RPH) requires the presence of a 19-nucleotide inverted repeat sequence upstream of the gene, known as the RAE. While orthologous RPH have been identified in many Gram-positive bacteria, the co-occurrence of RPH with its upstream RAE has been observed exclusively in Actinobacteria, but not in Bacilli. Consequently, RPH genes present in Bacilli may not be functionally active [73]. Indeed, to identify the presence of RAE, a BLAST analysis was performed on the upstream intergenic regions of the *rphB* genes in all three strains, and no RAE sequences were detected in any of the strains (data not shown). Furthermore, a study by Liu et al. investigated 96 *B. amyloliquefaciens* genomes deposited in the NCBI database with N50 sizes greater than 50 kb. The prevalence frequency of the *cfrB* gene was found to be 85.4%, while that of *satA* was 95.8%. Notably, the *rphB* gene was detected in all 96 genomes [74]. These results suggest that the three genes are more likely to represent intrinsic resistance determinants in *B. amyloliquefaciens*, rather than acquired antibiotic resistance elements [37]. Collectively, the absence of hemolytic activity, the lack of significant antibiotic resistance, and the absence of virulence factors in *B. amyloliquefaciens* EG025 reinforces its suitability for probiotic use.

Finally, full-genome based phylogenetic analyses were conducted using PyANI and CVtree to verify the genetic distances between *B. amyloliquefaciens* EG025 and other strains. Both tools analyze complete genome sequences, including non-coding regions, thereby yielding a refined classification even within a single species [75]. In this result, the PyANI-based analysis revealed that the 103 strains clustered into several groups according to their ANI values, with strain RD7-7 exhibiting the highest ANI (98.877%) and strain CUN9 the lowest (94.046%). In addition, strains RD7-7, 35M, Ba13, Bam1, H, PP19, TA208, XH7, SRCM124317, and LL3 exhibited higher ANI values relative to EG025, confirming their relatively close genetic relationship. Similarly, the CVtree analysis demonstrated that strains RD7-7, Ba13, 35M, YP6, PP19, MT45, XH7, TA208, LL3, H, Bam1, and SRCM124317 formed proximate branches with *B. amyloliquefaciens* EG025. Although EG025 consistently clustered with similar groups in both analyses, slight differences were observed. Specifically, while the 20 strains most closely related to EG025 by PyANI were also near EG025 in CVtree, strains Fad77 and Fad108 formed relatively distant branches in CVtree analysis. Conversely, strains SRCM101267 and SRCM123386 were closely clustered with EG025 in CVtree yet showed ANI values of 97.843% and 97.842% in PyANI analysis, indicating a more distant genetic relationship. These discrepancies are likely attributable to methodological differences. PyANI analysis employs pairwise alignment-based methods using BLAST, MUMmer, and NUCmer, whereas CVtree uses an alignment-free, k-mer-based approach [76, 77]. Moreover, it has been reported that for two strains to be defined as identical, strains must share an ANI of at least 99.99% and over 99.0% gene content [78]. In the current analysis, the RD7-7 strain exhibited an ANI value of 98.88% relative to EG025. This result indicates that a noticeable genetic difference exists between EG025 and RD7-7, thereby substantiating that EG025 represents a novel strain.

Taken together, the experimental and genomic findings strongly support the therapeutic probiotic potential of *B. amyloliquefaciens* EG025 as an alternative treatment approach for celiac disease. However, the current study has several limitations that must be addressed before EG025 can be practically applied as a probiotic therapeutic. While the experimental and genomic data presented are promising, the underlying molecular mechanisms of EG025’s gliadin-degrading activity remain unclear. Additional protein expression analyses, proteomic profiling, and comprehensive omics studies such as transcriptomics and metabolomics are necessary to elucidate these mechanisms and thoroughly map the relevant metabolic pathways.

In addition, functional validation of specific probiotic-related genes identified through genomic analysis would be beneficial. Employing techniques such as CRISPR-Cas-mediated genome editing and homologous recombination could enable precise manipulation and enhancement of desired probiotic characteristics or gliadin-degrading capabilities. Such approaches could also help confirm the specific roles of identified genes and clusters in probiotic functionalities.

Moreover, the reliance of this study on in vitro assessments limits the direct extrapolation of results to in vivo conditions. Animal model studies are essential to further evaluate potential health impacts, validate therapeutic efficacy, confirm safety profiles, and assess the strain's long-term stability and colonization efficiency within the gastrointestinal tract. Conducting these studies will provide comprehensive insights into the practical utility of *B. amyloliquefaciens* EG025 and facilitate its development into an effective probiotic treatment option for celiac disease.

## Conclusion

This study systematically evaluated *B. amyloliquefaciens* EG025 as a probiotic candidate for celiac disease through combined phenotypic and genomic analyses. *B. amyloliquefaciens* EG025 demonstrated strong potential as a targeted probiotic intervention for celiac disease. The strain not only hydrolyzed gliadin efficiently under neutral and alkaline pH conditions but also recovered over 90% of its gliadin-degrading activity after transient exposure to acidic environments, highlighting its resilience to gastric stress. When challenged with simulated gastric juice and bile salts, *B. amyloliquefaciens* EG025 maintained viability above 80% for up to 6 h and retained approximately 61% viability even after 12 h in bile, confirming its capacity to survive the journey through the human gastrointestinal tract. Moreover, phenotypic hemolytic activity and significant antibiotic resistance were not detected, indicating a lack of concerning harmful traits.

Genomic analyses revealed a repertoire of probiotic-relevant genes, including those encoding chaperone proteins, bile salt hydrolases, and other stress-response factors, alongside biosynthetic clusters for essential vitamins and a suite of antimicrobial secondary metabolites such as PKS, terpenes, NRPS, and lanthipeptides. Crucially, no mobile elements associated with antibiotic resistance or virulence factors were detected, underscoring a clean safety profile. Phylogenomic analysis confirmed genetic novelty of *B. amyloliquefaciens* EG025, with average nucleotide identity values below 99% compared to its closest known strains.

Collectively, these findings indicate that *B. amyloliquefaciens* EG025 holds significant potential as a probiotic candidate and may offer therapeutic benefits for celiac disease treatment. However, detailed mechanistic studies including proteomic, transcriptomic, and metabolomic analyses as well as functional validation of key probiotic and gliadin-degrading genes are needed to fully elucidate *B. amyloliquefaciens* EG025’s mode of action and ensure its efficacy. Additionally, further in vivo evaluation in appropriate animal or humanized models is warranted to assess gastrointestinal colonization, immunomodulatory effects, long-term safety, and to optimize formulation for clinical application.

## Supplementary Information

Below is the link to the electronic supplementary material.

ESM 1 (DOCX 1.99 MB)

## Data Availability

The genome sequence of *B. amyloliquefaciens* EG025 is available in the NCBI Genome database under the accession number CP187241 (BioProject: PRJNA1247436; BioSample: SAMN47812635).
