# Supplementary material for: Genomic and Phenotypic Evaluation of the Gliadin-Degrading Probiotic Bacillus amyloliquefaciens EG025 from Cheonggukjang for Celiac Disease Treatment
Source: Probiotics Antimicrob Proteins. 2025 Sep 2;18(3):4012–36. doi: 10.1007/s12602-025-10728-7 (PMC13176060; doi:10.1007/s12602-025-10728-7)
Supplement: Supplementary file 1 — (DOCX 1.99 MB) [file 12602_2025_10728_MOESM1_ESM.docx]

**Supplementary data**

**Title: Genomic and Phenotypic Evaluation of the Gliadin Degrading Probiotic *Bacillus amyloliquefaciens* EG025 from Cheonggukjang for Celiac Disease Treatment**

**Jinchul Jo^1,^****^2†^, Seoae Cho^2^, Heebal Kim^1,2*^**

^1^Department of Agricultural Biotechnology and Research Institute of Agriculture and Life Sciences, Seoul National University, Seoul, Republic of Korea

^2^eGnome Inc, Seoul, Republic of Korea

†This author contributed as the first author of this study.

**Journal name:**

Probiotics and Antimicrobial Proteins

*** Correspondence:**

**Heebal Kim, Ph.D.**

**E-mail: heebal@snu.ac.kr**

**Content in Supplementary data**

Table S1. Detected antibiotic resistance genes and virulence factors in *Bacillus amyloliquefaciens* EG025, LFB112, and TL106

Figure S1. AntiSMASH analysis of secondary metabolite biosynthetic gene clusters in the genome of *B. amyloliquefaciens* EG0

**Table S1. Detected antibiotic resistance genes and virulence factors in *Bacillus amyloliquefaciens* EG025, LFB112, and TL106**

| ***Bacillus amyloliquefaciens* EG025** | | | | | | | | | | |
| --- | --- | --- | --- | --- | --- | --- | --- | --- | --- | --- |
| **Database** | **START** | **END** | **STRAND** | **GENE** | **COVERAGE** | **COVERAGE_MAP** | **GAPS** | **%IDENTITY** | **%COVERAGE** | **ACCESSION** |
| **ARG-ANNOT** | 3107719 | 3108240 | + | (AGly)satA | 1-522/522 | =============== | 0/0 | 86.78 | 100 | NG_064662:101_622 |
|  | 3110706 | 3111755 | + | (MLS)cfr(B) | 1-1050/1050 | =============== | 0/0 | 89.24 | 100 | KR610408:4995-6044 |
| **CARD** | 549761 | 552357 | + | rphB | 1-2648/2655 | ========/====== | 5/57 | 80.88 | 97.7 | KX531052.1:0-2655 |
|  | 3110706 | 3111755 | + | clbA | 1-1050/1050 | =============== | 0/0 | 93.62 | 100 | CP006845.1:539695-540745 |
| **MEGARES** | 549761 | 552361 | + | RPH | 1-2607/2607 | ========/====== | 7/26 | 81.85 | 99.39 | MEG_6086 |
|  | 3107719 | 3108240 | + | SAT | 1-522/522 | =============== | 0/0 | 86.78 | 100 | MEG_6164 |
|  | 3110706 | 3111755 | + | CLBA | 1-1050/1050 | =============== | 0/0 | 93.62 | 100 | MEG_1726 |
| **NCBI** | 549761 | 552361 | + | rphC | 1-2607/2607 | ========/====== | 7/26 | 81.85 | 99.39 | NG_063825.1 |
|  | 3107719 | 3108240 | + | satA_Bs | 1-522/522 | =============== | 0/0 | 86.78 | 100 | NG_064662.1 |
|  | 3110706 | 3111755 | + | clbA | 1-1050/1050 | =============== | 0/0 | 93.62 | 100 | NG_062350.1 |
| **PlasmidFinder** | Not detected | | | | | | | | | |
| **ResFinder** | 3110706 | 3111755 | + | cfr(B)_3 | 1-1050/1050 | =============== | 0/0 | 89.24 | 100 | KR610408 |
| **EcOH** | Not detected | | | | | | | | | |
| **ecoli_vf** | Not detected | | | | | | | | | |
| **VFDB** | Not detected | | | | | | | | | |
| ***Bacillus amyloliquefaciens* LFB112** | | | | | | | | | | |
| **Database** | **START** | **END** | **STRAND** | **GENE** | **COVERAGE** | **COVERAGE_MAP** | **GAPS** | **%IDENTITY** | **%COVERAGE** | **ACCESSION** |
| **ARG-ANNOT** | 557131 | 558166 | + | (MLS)cfr(B) | 1-1036/1050 | =============== | 0/0 | 87.64 | 98.67 | KR610408:4995-6044 |
|  | 2605521 | 2606042 | - | (AGly)satA | 1-522/522 | =============== | 0/0 | 84.87 | 100 | NG_064662:101_622 |
| **CARD** | 557131 | 558180 | + | clbA | 1-1050/1050 | =============== | 0/0 | 96.76 | 100 | CP006845.1:539695-540745 |
| **MEGARES** | 557131 | 558180 | + | CLBA | 1-1050/1050 | =============== | 0/0 | 96.76 | 100 | MEG_1726 |
|  | 2603935 | 2605311 | - | TETL | 1-1377/1377 | =============== | 0/0 | 86.64 | 100 | MEG_7102 |
|  | 2605521 | 2606042 | - | SAT | 1-522/522 | =============== | 0/0 | 84.87 | 100 | MEG_6164 |
| **NCBI** | 557131 | 558180 | + | clbA | 1-1050/1050 | =============== | 0/0 | 96.76 | 100 | NG_062350.1 |
|  | 1849157 | 1851753 | + | rphC | 1-2606/2607 | ========/====== | 4/23 | 80.37 | 99.35 | NG_063825.1 |
|  | 2603935 | 2605311 | - | tet(L) | 1-1377/1377 | =============== | 0/0 | 86.64 | 100 | NG_048204.1 |
|  | 2605521 | 2606042 | - | satA_Bs | 1-522/522 | =============== | 0/0 | 84.87 | 100 | NG_064662.1 |
| **PlasmidFinder** | Not detected | | | | | | | | | |
| **ResFinder** | 557131 | 558166 | + | cfr(B)_3 | 1-1036/1050 | =============== | 0/0 | 87.64 | 98.67 | KR610408 |
|  | 2603935 | 2605311 | - | tet(L)_5 | 1-1377/1377 | =============== | 0/0 | 86.64 | 100 | X08034 |
| **EcOH** | Not detected | | | | | | | | | |
| **ecoli_vf** | Not detected | | | | | | | | | |
| **VFDB** | Not detected | | | | | | | | | |
| ***Bacillus amyloliquefaciens* TL106** | | | | | | | | | | |
| **Database** | **START** | **END** | **STRAND** | **GENE** | **COVERAGE** | **COVERAGE_MAP** | **GAPS** | **%IDENTITY** | **%COVERAGE** | **ACCESSION** |
| **ARG-ANNOT** | 1355982 | 1356503 | + | (AGly)satA | 1-522/522 | =============== | 0/0 | 85.44 | 100 | NG_064662:101_622 |
|  | 3447576 | 3448611 | - | (MLS)cfr(B) | 1-1036/1050 | =============== | 0/0 | 87.64 | 98.67 | KR610408:4995-6044 |
| **CARD** | 3447562 | 3448611 | - | clbA | 1-1050/1050 | =============== | 0/0 | 96.76 | 100 | CP006845.1:539695-540745 |
| **MEGARES** | 1355982 | 1356503 | + | SAT | 1-522/522 | =============== | 0/0 | 85.44 | 100 | MEG_6164 |
|  | 1367114 | 1368490 | + | TETL | 1-1377/1377 | =============== | 0/0 | 86.86 | 100 | MEG_7102 |
|  | 3447562 | 3448611 | - | CLBA | 1-1050/1050 | =============== | 0/0 | 96.76 | 100 | MEG_1726 |
| **NCBI** | 1355982 | 1356503 | + | satA_Bs | 1-522/522 | =============== | 0/0 | 85.44 | 100 | NG_064662.1 |
|  | 1367114 | 1368490 | + | tet(L) | 1-1377/1377 | =============== | 0/0 | 86.86 | 100 | NG_048204.1 |
|  | 2087592 | 2090188 | - | rphC | 1-2606/2607 | ========/====== | 5/23 | 80.37 | 99.35 | NG_063825.1 |
|  | 3447562 | 3448611 | - | clbA | 1-1050/1050 | =============== | 0/0 | 96.76 | 100 | NG_062350.1 |
| **PlasmidFinder** | 6535 | 6728 | - | Col(SD853)_1 | 1-194/194 | =============== | 0/0 | 82.47 | 100 | NC_015392 |
|  | 15098 | 15291 | - | Col(SD853)_1 | 1-194/194 | =============== | 0/0 | 82.47 | 100 | NC_015392 |
| **ResFinder** | 1367114 | 1368490 | + | tet(L)_5 | 1-1377/1377 | =============== | 0/0 | 86.86 | 100 | X08034 |
|  | 3447576 | 3448611 | - | cfr(B)_3 | 1-1036/1050 | =============== | 0/0 | 87.64 | 98.67 | KR610408 |
| **EcOH** | Not detected | | | | | | | | | |
| **ecoli_vf** | Not detected | | | | | | | | | |
| **VFDB** | Not detected | | | | | | | | | |


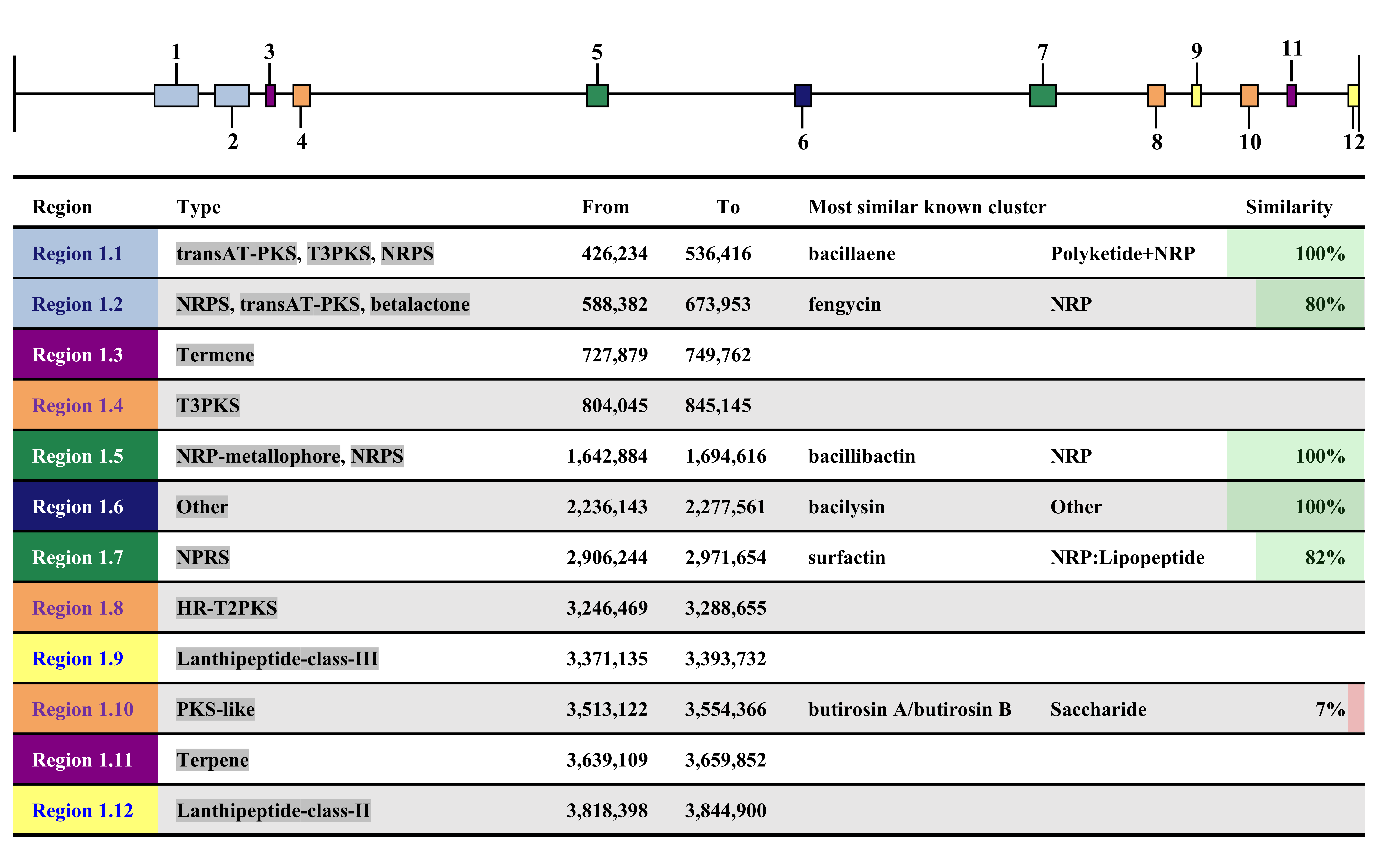
 **Figure S1. AntiSMASH analysis of secondary metabolite biosynthetic gene clusters in the genome of *B. amyloliquefaciens* EG025.** The genome of *B. amyloliquefaciens* EG025 was found to harbor 12 biosynthetic gene clusters, including those for polyketide synthase (PKS), terpene, non-ribosomal peptide synthetase (NRPS), and lanthipeptide. Among these, the gene clusters responsible for the biosynthesis of bacillaene, bacillibactin, and bacilysin exhibited 100% similarity to previously characterized clusters.
